# Budget impact analysis of continuous glucose monitoring in individuals with type 2 diabetes on insulin treatment in England

**DOI:** 10.1186/s13561-024-00505-7

**Published:** 2024-05-06

**Authors:** Murtada Alsaif, Ali Farhat, Zoe Blumer, Leela Barham

**Affiliations:** 1 IPG Health Global Market Access, London, UK; 2 PharmaSaif Ltd, Slough, UK; 3 Learna Ltd in partnership with the University of South Wales, Cardiff, Wales, UK

**Keywords:** Adult, Insulin, Blood Glucose, Diabetes Mellitus, Type 2, Blood Glucose Self-Monitoring, COVID-19, Hypoglycemia, Insulin, Regular, Human, Diabetic Ketoacidosis, Decision Making

## Abstract

**Introduction:**

In 2022, updated guidance from NICE expanded the options for self-monitoring of blood glucose for patients with type 2 diabetes (T2DM), to include continuous glucose monitoring (CGM). In this budget impact analysis, the cost impact of CGM was compared with traditional self-monitoring of blood glucose (SMBG) in adults with T2DM over 1 year from the commissioner perspective in England.

**Research Design and methods:**

The NICE-eligible T2DM cohort was split into 4 subgroups to enable nuanced costing by insulin administration frequency: basal human insulin, premixed insulin, basal-bolus insulin and bolus insulin. The model’s cost components comprised mild and severe hypoglycaemia (SH), diabetic ketoacidosis (DKA), consumables and healthcare resource utilisation in primary and secondary care.

**Results:**

The introduction of CGM is estimated to be cost additive by approximately £4.6 million in the basecase, driven by increased spending on the CGM device. Overall, healthcare activity was reduced by approximately 20,000 attendances, due to fewer SH and DKA episodes in the CGM arm. General Practitioner (GP) practice-based activity is expected to drop after the first year as patients requiring CGM training is reduced. The budget impact could be neutralised if the CGM sensor was discounted by 13.2% (£29.76 to £25.83).

**Conclusions:**

CGM may result in increased spending in the NICE-eligible T2DM cohort but is expected to reduce demand on secondary care services and GP time. These findings may be of interest to local decision-makers who wish to resolve the COVID-19 backlog with transformational investment in primary care to reduce secondary care activity.

**Supplementary Information:**

The online version contains supplementary material available at 10.1186/s13561-024-00505-7.

## Background

Type 2 diabetes mellitus (T2DM) affects 6.66% of adults in England [1], a third of whom, will have at least one microvascular complication when diagnosed [2]. Analysis suggests that better glucose and glycated haemoglobin (HbA1c) management could avoid 80% of the £10 billion spent on diabetes each year [2].

Even though several classes of drugs are recommended ahead of insulin to treat patients with T2DM [3], the progressive nature of the disease and the decline of endogenous insulin over time eventually necessitates the use of insulin in a proportion of people with T2DM [4]. Hypoglycaemia is an established adverse effect of using insulin and it is defined as “a lower than normal blood-glucose concentration” [5].

Since 2015, NICE recommended all T2DM patients on insulin self-monitor their glucose levels with traditional strips and lancets [3]. In 2022, the NICE-recommended monitoring options expanded to include continuous glucose monitoring (CGM) [3]. The CGM device is applied to the patient’s arm, providing interstitial glucose readings on demand for up to 14 days, in some devices [6].

Evaluation and endorsement of medical devices from NICE is seen as a way to make the case for reimbursement easier, but is not mandatory. Manufacturers, or others, can inform NICE of a device. NICE uses different programmes to evaluate devices, ranging from a briefing that summarises key clinical evidence and economic models through to a NICE guideline that will include a systematic review of the clinical evidence and economic modelling. Cost consequence analysis is used for clinically non-inferior technologies and cost-effectiveness analysis for clinically superior technologies [7].

In a systematic review, which included 12 studies (2,173 diabetes patients on insulin; 1,663 using CGM vs. 510 using self-monitoring of blood glucose, SMBG), CGM was associated with a reduction from baseline in [6]:

- HbA1c of -0.26% (-3 mmol/mol) (95% CI -0.43 to -0.09).
- The time at risk of hypoglycaemia (blood glucose < 70 mg/dL); -0.60 h/day (95% CI -1.18 to -0.03), although there was no change in the frequency of hypoglycaemia.

To date, there have been several budget impact analyses (BIA) that compare the difference in cost for monitoring blood glucose with traditional strips and lancets versus CGM. These analyses considered patients with type 1 diabetes mellitus (T1DM) in Canada [8], England/UK [9–12], Spain [13] and the USA [14], T2DM on basal-bolus insulin treatment in Spain [15], patients with diabetes on intensive insulin treatment in the USA [16] and T1DM and T2DM patients receiving multiple daily insulin in Argentina [17].

NICE CGM reimbursement criteria for “adults (aged 18 years or over) with type 2 diabetes on multiple daily insulin injections” include but are not limited to recurrent hypoglycaemia or severe hypoglycaemia, impaired hypoglycaemia awareness, or if ≥ 8 self-measurements are required each day [3]. This research aims to compare CGM with SMBG in the NICE-eligible T2DM in England, and quantify the budget impact over 1 year. The model takes the perspective of the NHS commissioner.

## Methods

This de-novo model was constructed in line with guidance from the ISPOR Task Force [18]. The model structure and inputs were guided by previously published cost analyses [1, 4, 12, 13, 15, 16].

The modelled population was estimated using the NICE Resource Impact template [1]. Patients who administer insulin at greater frequencies require more frequent glucose testing [19] and are at greater risk of hypoglycaemia. These patients incur a greater cost to the commissioner. The NICE patient funnel did not consider patients on different insulin regimens who generate differential costs to the commissioner. Therefore, data from a retrospective analysis were used to split the NICE-eligible population into 4 subgroups to enable nuanced costing: twice-daily basal human insulin (9.3%), twice-daily premixed insulin (62.5%), ≥three times a day basal-bolus insulin (25.7%) and ≥ three times a day bolus insulin (2.5%) [19]. A detailed breakdown of the patient funnel can be found in the supplementary Appendix. Please note that T2DM patients on bolus insulin are expected to be a very small group, potentially smaller in current practice, compared to the references used to derive the 2.5% above. However, we could not find any data to justify their exclusion, and they could reasonably be expected to disproportionately benefit from the use of CGM.

Within the model, 8.6% of CGM patients discontinued use, based on the results of the 12 month extension to the pivotal study for CGM, which showed that 12 of 139 patients discontinued their use of CGM due to adverse events; ‘other’ reasons, physician decision or withdrawal from the study [20].

Patients who discontinue CGM were modelled to go back to using traditional strips and lancets, in line with NICE recommendations [3], and the basecase assumed this occurred on day 28 (2 sensors used). In the BIA, patients who discontinue treatment were costed as per CGM for 28 days and as per SMBG for 337 days. Costs for adverse events were not included, we assumed that adverse events simply lead to discontinuation and incurred no further costs.

**Fig. 1:**
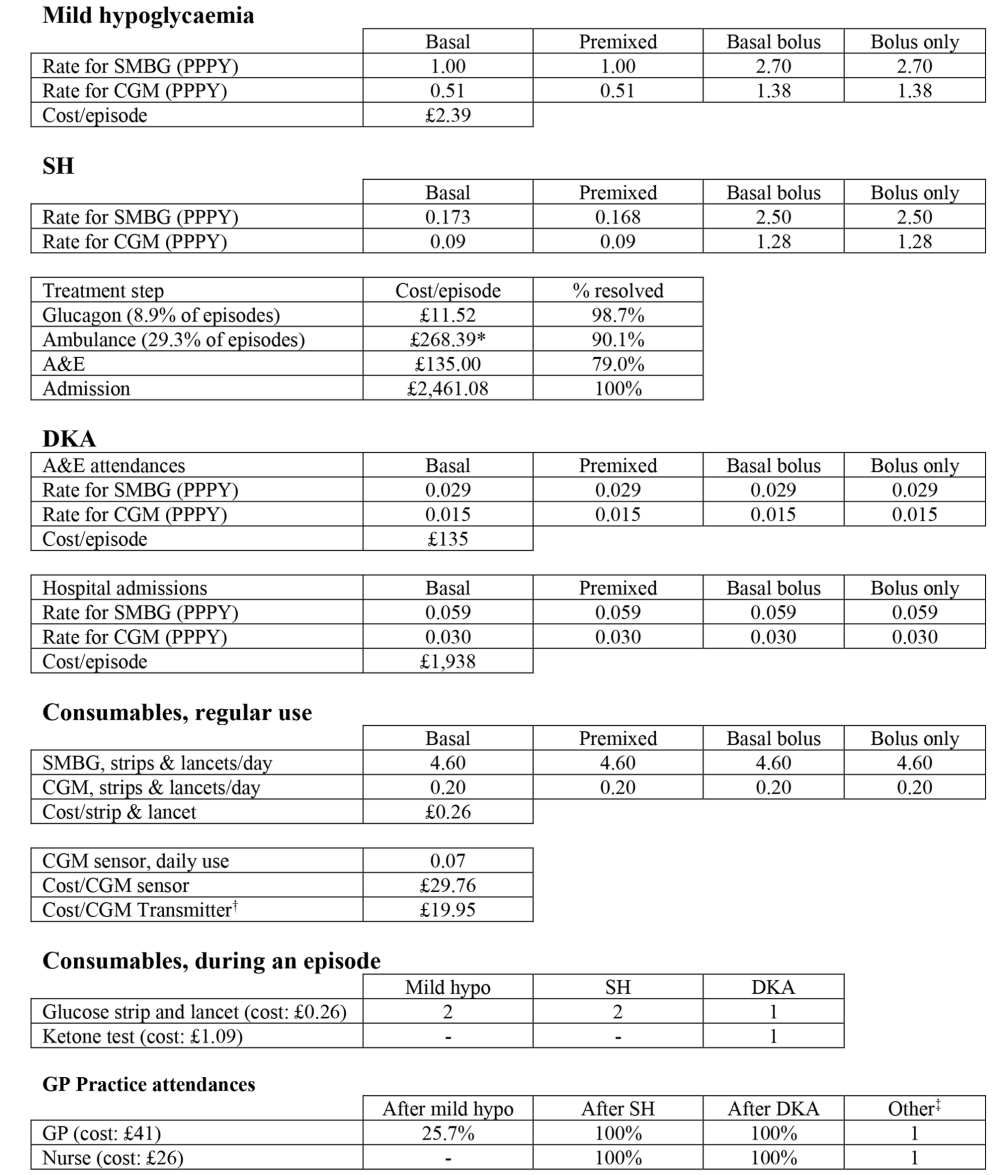
Summary of costs modelled. *Ambulance attendance costs £268.39 if SH resolved or £390.08 if SH not resolved. †Paid for once per year for each CGM patient. ‡ 1 annual review with a GP and 1 CGM training session with a practice nurse. A&E: Accident & Emergency, CGM: continuous glucose monitoring, DKA: diabetic ketoacidosis, GP: general practitioner, hypos: hypoglycaemias, PPPY: per patient per year, SH: severe hypoglycaemia, SMBG: self-monitoring of blood glucose

The model accounted for consumables for glucose and ketone monitoring, healthcare resource utilisation and outcomes that can be directly impacted by the use of CGM; mild hypoglycaemia, severe hypoglycaemia (SH) and diabetic ketoacidosis (DKA) [16]. The budget impact of using CGM is driven by differing use of consumables, healthcare utilisation and the reduction of hypoglycaemia and DKA events with CGM compared to SMBG. The costs modelled are summarised in Fig. 1.

Cost offsets from HbA1c improvements with CGM were not included in this model [3].

### Mild hypoglycaemia

Mild hypoglycaemia is defined as blood glucose < 3.5 mmol/L in conscious patients or when a conscious patient experiences hypoglycaemia symptoms [21]. In these cases, patients in England are advised to consume sugar, including 1 to 2 tubes of glucose gel [21].

The reported median incidence of mild self-reported hypoglycaemia is 1 per patient per year (PPPY) (range: 0 to 44) in T2DM insulin users < 2 years and 2.7 PPPY (range: 0 to 144) in T2DM insulin users > 5 years [22]. NICE have recommended initiating insulin therapy in T2DM with either basal insulin or premixed insulin. This can be escalated to a basal-bolus regime “if blood glucose control remains inadequate” [3]. Therefore, it is *assumed* that the median hypoglycaemia incidence rate for users of insulin < 2 years applies to basal insulin and premixed insulin users, whereas the rate for users of insulin > 5 years applies to basal-bolus and bolus only users in the model. The rationale being that patients would need time to be escalated to higher doses of insulin or higher insulin injection frequency. For all regimens, the reduction in mild hypoglycaemia with CGM was -48.8%, as per the peer-reviewed T2DM BIA based in Spain [15].

The cost of treating mild hypoglycaemia to the payer in England was based on prescribed glucose 40% oral gel. No reports were found to inform the proportion of mild hypoglycaemia episodes that were treated with glucose 40% oral gel. In this analysis, all episodes were modelled to be treated with 1 × 25 g tube of glucose 40% oral gel. The referenced justification for this can be found in the Appendix.

The Drug Tariff cost of 75 g Glucose 40% oral gel (3 × 25 g tubes) was £7.16 [23]. The model assumed 1 tube [21] resolves mild hypoglycaemia, therefore, the cost per episode was £2.39.

### Severe hypoglycaemia (SH)

SH is defined as hypoglycaemia that requires the assistance of someone else [11, 13, 15, 16].

The modelled annual incidence of SH in the basal insulin group was 0.173 PPPY, pre-mixed 0.168 PPPY, basal-bolus 2.5 PPPY and bolus only 2.5 PPPY. The referenced justification for this can be found in the Appendix. For all regimens, the reduction in SH with CGM used was -48.8% [15].

**Fig. 2:**
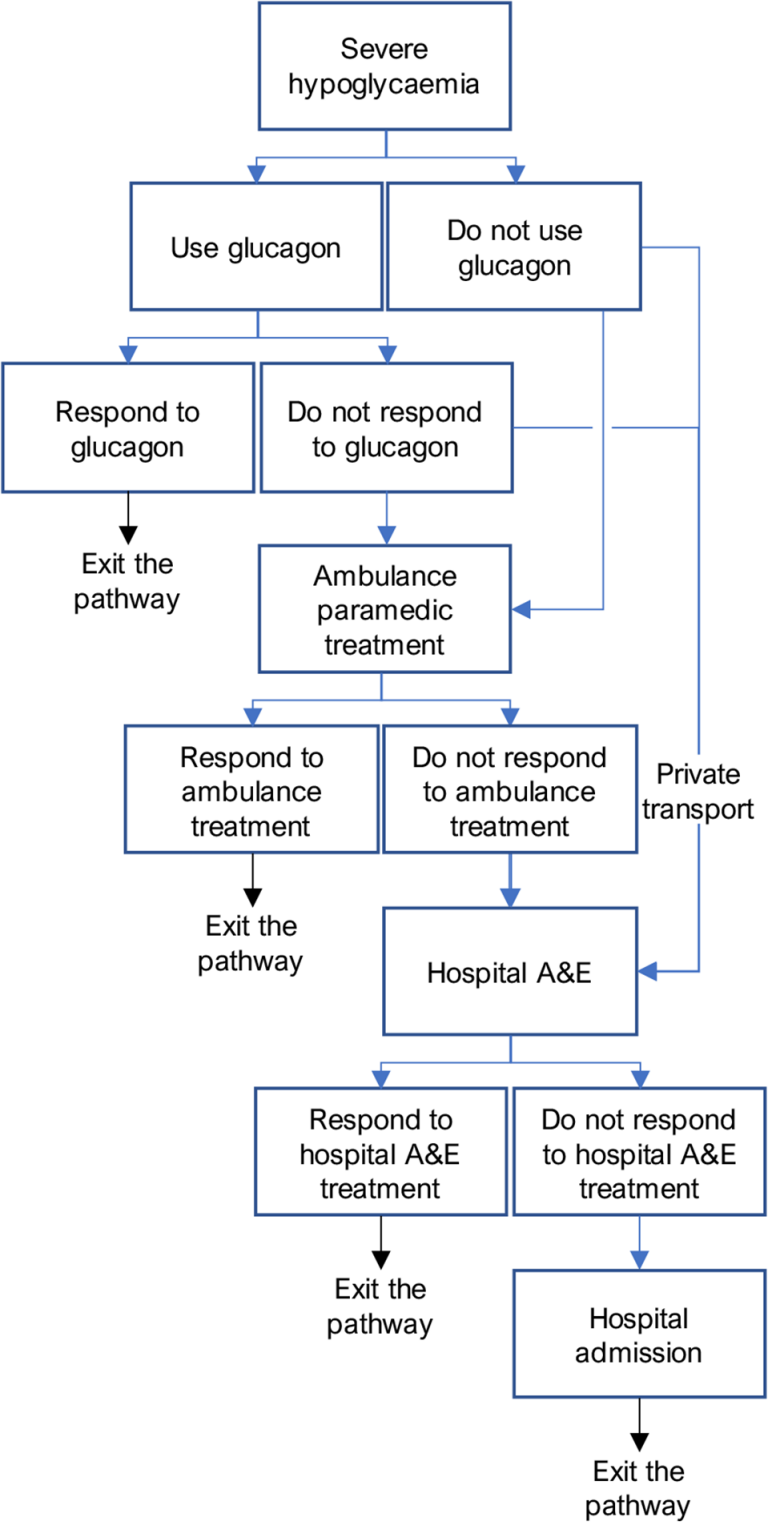
Severe hypoglycaemia treatment pathway. A&E: Accident & Emergency

SH was modelled with several steps in its treatment pathway (Fig. 2), in an attempt to adhere to currently reimbursed treatment in England, following recommendations for patient care and was informed by previous publications [4, 24].

The first step of the pathway was treatment at home by a trained friend or family member with 1 x glucagon 1 mg injection [21]. No literature was found to inform the rate of glucagon use in SH. A rate of usage of 8.9% was derived. The referenced justification for this can be found in the Appendix. SH resolution was modelled to occur in 98.7%, which was the average response rate from two clinical trials evaluating glucagon 1 mg pre-filled injections [25].

The cost of a single vial of glucagon 1 mg was £11.52 [23]. An additional glucagon product is licensed in the UK; Baqsimi 3 mg nasal powder in single-dose container [26]. However, this product was not registered in the Dictionary of Medicines and Devices at the time of writing, as such it was assumed to be unavailable for prescribing and was excluded from the model [27].

In the second step of the pathway, ambulance treatment is included in 29.3% of SH cases [24]. Ambulance treatment was modelled to resolve SH in 90.1% of cases, see the Appendix for how this was derived, at a cost of £268 [28]. If SH episodes were not resolved, patients were modelled to be transported to hospital for further emergency treatment at a cost of £390 [28].

In step 3, patients who were not transported to hospital via ambulance are modelled to have arrived at hospital by private transport. This incurs no cost in the model. In a previous cost analysis of hypoglycaemia, 21% of patients with SH were admitted to hospital after treatment at Accident and Emergency (A&E) [4], consequently, the model utilises a rate of 79% for SH resolution in A&E. Treatment at A&E was costed as a Category 1 investigation with category 1–2 treatment (Healthcare Resource Group (HRG) code: VB09Z) [4] using the 2023/25 NHS Payment Scheme price; £135 [29].

In Step 4, SH episodes that are not resolved in A&E are escalated to a hospital admission, at a cost of £2,461. How this cost was derived can be found in the Appendix. All SH episodes that reached a hospital admission were modelled to be resolved.

### Diabetic ketoacidosis (DKA)

DKA is “the most common acute hyperglycaemic emergency in people with diabetes” [30]. England Hospital Episode Statistics (HES) data were used to estimate the cost of severe DKA in the NICE-eligible T2DM cohort. According to NHS Digital (2020) [31], there were 8,592 episodes for T2DM patients admitted with DKA (ICD-Code E11.1) as the Primary Diagnosis in 2019–2020 (this period aligns with the Office for National Statistics, ONS, population used in the patient funnel). The estimated number of DKA admissions that relate to the NICE-eligible population was derived as 2,880 (derivation detailed in the Appendix). DKA admissions in the NICE-eligible population were subsequently split across the four regimens, per the patient funnel.

HES data report a 4,207 subset of 8,592 finished consultant episodes as “Emergency” [31]. It is assumed that these admissions resulted from an A&E attendance, and this was included in the BIA. As above, the 4,207 subset was further broken down to derive attendances relevant for the NICE-eligible population; an estimated 1,410 A&E attendances (detailed in the Appendix). The rate of reduction in DKA episodes with CGM was 48.8% reduction, in line with hypoglycaemia.

The cost of DKA at A&E was assumed to be the same as SH; £135, and the cost of a DKA admission was derived as £1,938 (detailed in the Appendix). It is assumed that all DKA hospital admissions are resolved.

Less severe DKA episodes managed outside of hospitals could not be explored for lack of data.

### Consumables

Patients in both scenarios (SMBG and CGM) were modelled to continue using traditional strips and lancets [32], in line with NICE guidance [3]. However, the frequency of daily testing differed. The annual cost of SMBG testing in patients using traditional strips and lancets was reported as £436.54 with a cost per test of £0.26 for a strip and lancet [3], amounting to 4.6 tests per day. Each patient was modelled to receive one CGM transmitter that was assumed to last the full model time horizon (1 year) and this was costed at £19.95 [23]. Over the year, each CGM patient was modelled to use one CGM sensor, costing £29.76 [23], every 14 days [33]. Furthermore, CGM patients were modelled to test with traditional strips and lancets 0.2 times per day [20].

Following recommendations in England [21], SMBG patients were modelled to test with 2 strips and lancets after a mild hypoglycaemia episode. In contrast, CGM patients incurred no extra cost as it was assumed they would scan their glucose levels more frequently, rather than using more strips and lancets. CGM patients who discontinue CGM and return to SMBG were costed as SMBG patients. SH episodes treated with glucagon or by Ambulance teams were modelled to receive the same enhanced testing with traditional strips and lancets in the SMBG group. To align with DKA recommendations in England (blood glucose test followed by a ketone test if glucose levels exceed 11 mmol/L [34]), one blood glucose and one ketone test was costed for SMBG patients, and one ketone test was costed for CGM patient for every DKA admission. The cost of each ketone test was derived as £1.09 (detailed in the Appendix).

### General practitioner (GP) costs

All patients were modelled to receive a single annual review with a GP. It was assumed that the use of CGM did not add extra time to a typical GP annual review. With CGM, a 30 min training consultation was modelled to occur with a Practice Nurse as “CGM should be provided by a team with expertise in its use, as part of supporting people to self-manage their diabetes” [3]. A mild hypoglycaemic episode was modelled to trigger a GP attendance in 25.7% of episodes [4].

Following SH [35] or DKA [36], patients were modelled to receive a 30-min consultation with a Practice Nurse to receive structured education in addition to a medication review with a GP.

A GP appointment was costed at £41 [37] and a Practice Nurse session at £26 (assuming a 30-min session and an hourly rate of £52 per hour) [37].

### Scenario analysis

The scenario analysis took the perspective of the budget holder, replacing basecase inputs with other plausible inputs. All scenarios tested are presented in Table 1.

The model was built in Microsoft Excel and this research was deemed as low risk and as such was reviewed by the Low Risk Ethical procedure at the Faculty of Life Science and Education, University of South Wales and granted approval.

**Table 1:** Scenarios tested in the BIA

Scenario	Change	Explanation
1	No hypoglycaemia or DKA with CGM	Newer versions of the CGM sensor device provide alerts of impending hypo- or hyperglycaemia. Therefore, they can potentially prevent all episodes of hypoglycaemia and DKA. In this scenario, all inputs were kept the same as the basecase except CGM patients had no hypoglycaemia or DKA episodes.
2	Higher SMBG testing frequency	This was based on the pivotal trial extension study for CGM in T2DM patients, where the average sensor-scanning frequency was 7.1 times per day (median 5.7) [20].
3	Lower SH Hospital admission rate	Some patients require hospital admission to resolve their hypoglycaemia. In the Spanish BIA, this was set to 15.6% of A&E cases [15].
4	Highest admission cost	According to the National Tariff, the cost of admission with a CC score of 8 + for SH and DKA is £3,901 and £4,138 [29].
5	Lowest admission cost	According to the National Tariff, the cost of admission with a CC score of 0–1 for SH and DKA is £570 and £934 [29].
6	Higher rates for mild hypoglycaemia	The basecase utilised the median rates reported by the UK Hypoglycaemia Study Group [22]. In this scenario, the mean rates for T2DM insulin users < 2 years and > 5 years were used; 4.08 PPPY for basal and premixed and insulin users, 10.2 PPPY for basal-bolus and bolus insulin users [22].
7	More expensive glucagon injection	Here, the cost of the more expensive Ogluo 1 mg/0.2 ml pre-filled pens was used; £73.00 [23, 27], rather than the less expensive Drug Tariff price; £11.52 [23]
8	Lower rate of glucagon use	Glucose oral gel prescribing exceeds mild hypoglycaemias by 19 times. It is reasonable to assume the same applies to glucagon prescribing for SH. In this scenario, usage frequency was reduced by 19 times (from 8.9–0.5%).
9	Most favourable CGM conditions	Combination of inputs from scenarios 1 (No hypoglycaemia or DKA with CGM), 2 (Higher SMBG testing frequency), 4 (Highest admission cost) and 6 (Higher rates for mild hypoglycaemia).

*A&E: Accident & Emergency, CC score: complexity and comorbidity score, CGM: continuous glucose monitoring, DKA: diabetic ketoacidosis, SMBG: self-monitoring of blood glucose, SH: severe hypoglycaemia, T2DM: type 2 diabetes mellitus*

## Results

### Modelled population

Table 2 presents the patient funnel used in the model to estimate the NICE-eligible population of 48,797.

**Table 2:** Estimate of NICE-eligible patients to include in BIA

	Proportion	Number	Reference
England population in 2020	-	56,550,138	[38]
Adults (18 years and over)	78.6%	44,456,850	[38]
Prevalence of diabetes mellitus	7.4%	3,298,698	[1]
Proportion that are T2DM	90.0%	2,968,828	[1]
Multiple daily insulin injections users	3.55%	105,393	[1]
Proportion with impaired awareness, recurrent hypoglycaemia or SH	41.30%	43,527	[1]
Proportion that test ≥ 8 times/day	5.00%	5,270	[1]
**NICE-eligible population**	**-**	**48,797**	**-**
Basal only (human insulin)	9.3%	4,538	[19, 27]
Pre-mixed	62.5%	30,501	[19]
Basal-bolus	25.7%	12,557	[19]
Bolus only	2.5%	1,202	[19]

SH: severe hypoglycaemia, T2DM: type 2 diabetes mellitus

### Basecase cost outcomes

**Fig. 3:**
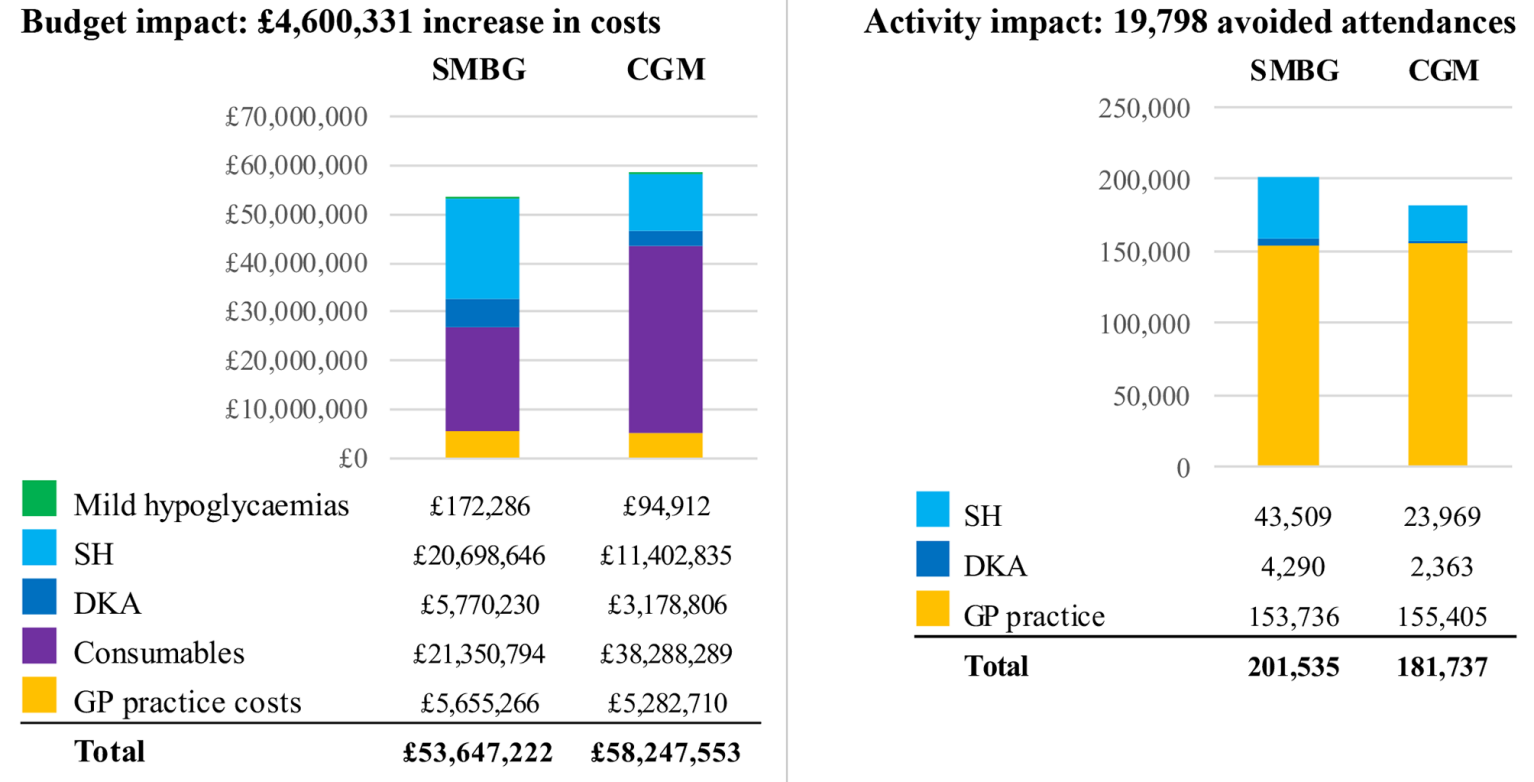
Overall budget and activity impact. CGM: continuous glucose monitoring, DKA: diabetic ketoacidosis, GP: general practitioner, hypos: hypoglycaemias, SH: severe hypoglycaemia, SMBG: self-monitoring of blood glucose

In this analysis, the cost of the CGM sensor drove a net cost increase of £4,600,331 but led to reduced healthcare activity by 19,798 attendances (Fig. 3). Cost savings with CGM can be seen for all items included, except consumables, which drive the overall cost increase. The reductions in costs are driven by reduced SH and DKA activity, and less costly interactions with GP staff. Detailed outcomes can be reviewed in Table 3.

**Table 3:** Detailed Basecase output for the overall cost outcomes, and primary vs. secondary care split

**Overall SMBG totals**	**Basal only**	**Premixed**	**Basal-bolus**	**Bolus only**	**Total**
Mild hypoglycaemias	£10,830	£72,795	£80,914	£7,747	£172,286
SH	£403,062	£2,634,880	£16,117,536	£1,543,168	£20,698,646
DKA	£536,580	£3,606,691	£1,484,798	£142,162	£5,770,230
Consumables	£1,983,753	£13,334,022	£5,505,862	£527,157	£21,350,794
GP practice costs	£304,397	£2,036,342	£3,024,908	£289,619	£5,655,266
Total	£3,238,622	£21,684,729	£26,214,019	£2,509,853	£53,647,222
**Primary care SMBG totals**	**Basal only**	**Premixed**	**Basal-bolus**	**Bolus only**	**Total**
Mild hypoglycaemias	£10,830	£72,795	£80,914	£7,747	£172,286
SH - Glucagon	£808	£5,282	£32,307	£3,093	£41,490
Consumables	£1,983,753	£13,334,022	£5,505,862	£527,157	£21,350,794
GP practice costs	£304,397	£2,036,342	£3,024,908	£289,619	£5,655,266
Total	£2,299,788	£15,448,440	£8,643,992	£827,616	£27,219,836
**Secondary care SMBG totals**	**Basal only**	**Premixed**	**Basal-bolus**	**Bolus only**	**Total**
SH - Ambulance	£58,822	£384,528	£2,352,156	£225,206	£3,020,713
SH - Hospital A&E	£71,128	£464,977	£2,844,262	£272,323	£3,652,691
SH - Hospital admission	£272,304	£1,780,092	£10,888,810	£1,042,546	£13,983,752
DKA - Hospital A&E	£17,701	£118,977	£48,980	£4,690	£190,348
DKA - Hospital admission	£518,879	£3,487,713	£1,435,818	£137,472	£5,579,883
Total	£938,834	£6,236,289	£17,570,027	£1,682,237	£26,427,386
**Overall CGM totals**	**Basal only**	**Premixed**	**Basal-bolus**	**Bolus only**	**Total**
Mild hypoglycaemias	£5,966	£40,103	£44,575	£4,268	£94,912
SH	£222,046	£1,451,549	£8,879,113	£850,128	£11,402,835
DKA	£295,601	£1,986,917	£817,972	£78,316	£3,178,806
Consumables	£3,560,336	£23,931,247	£9,853,305	£943,402	£38,288,289
GP practice costs	£369,225	£2,476,451	£2,224,089	£212,945	£5,282,710
Total	£4,453,174	£29,886,266	£21,819,054	£2,089,058	£58,247,553
**Primary care CGM totals**	**Basal only**	**Premixed**	**Basal-bolus**	**Bolus only**	**Total**
Mild hypoglycaemias	£5,966	£40,103	£44,575	£4,268	£94,912
SH - Glucagon	£445	£2,910	£17,798	£1,704	£22,857
Consumables	£3,560,336	£23,931,247	£9,853,305	£943,402	£38,288,289
GP practice costs	£369,225	£2,476,451	£2,224,089	£212,945	£5,282,710
Total	£3,935,973	£26,450,710	£12,139,768	£1,162,318	£43,688,768
**Secondary care CGM totals**	**Basal only**	**Premixed**	**Basal-bolus**	**Bolus only**	**Total**
SH - Ambulance	£32,405	£211,836	£1,295,797	£124,066	£1,664,104
SH - Hospital A&E	£39,184	£256,155	£1,566,897	£150,022	£2,012,259
SH - Hospital admission	£150,011	£980,649	£5,998,620	£574,336	£7,703,616
DKA - Hospital A&E	£9,751	£65,544	£26,983	£2,583	£104,862
DKA - Hospital admission	£285,849	£1,921,373	£790,989	£75,733	£3,073,944
Total	£517,201	£3,435,557	£9,679,286	£926,740	£14,558,785

A&E: Accident & Emergency, CGM: continuous glucose monitoring, DKA: diabetic ketoacidosis, GP: general practitioner, hypos: hypoglycaemias, SH: severe hypoglycaemia, SMBG: self-monitoring of blood glucose

In a breakeven analysis, the price of the CGM sensor would have to be discounted from £29.76 to £25.83 (13.2% discount) for CGM adoption to be cost neutral with the SMBG scenario.

In the CGM arm, costs increased by £16,468,932 in primary care but reduced by £11,868,601 in secondary care, as shown in the Appendix.

### Basecase activity outcomes

Secondary care activity was found to be reduced across the board. In primary care, attendances are marginally increased in GP practices, driven by an increase in attendances with practice nurses to train patients on using CGM. It is expected that this would only be needed once for patient initiation and would not be incurred in subsequent years. Additionally, attendances with GPs are reduced by 25% (fewer medication reviews after hypoglycaemia and DKA episodes). In GP practices, Commissioner spending is reduced by £372,556 (down from £5,655,266), despite an increase in total attendances. This is due to an increase in practice nurse attendances but a fall in more costly GP attendances. Detailed activity outcomes can be reviewed in the Appendix.

To understand the budget impact in the absence of nurse-led CGM training or in subsequent years of CGM implementation, the model was run without training attendances with practice nurses. Here, the total CGM activity dropped by 34% (from 201,535to 132,940 attendances). The total budget impact was marginally reduced.

### Drivers of cost outcomes in the basecase scenario

In the NICE-eligible population, the total modelled cost for SH and DKA in the SMBG arm was £26,468,876. This was reduced to £14,581,641 with CGM. Hospital admissions formed the largest portion of the costs for both the SMBG and CGM arms, albeit the activity component of SH was the smallest in the SMBG group. This was driven by the cost of admissions which is at least one order of magnitude greater than all other unit costs in the SH pathway. This is detailed in the Appendix.

In a breakeven analysis, the regular daily testing with strips and lancets would need to be performed 5.68 times per day in the SMBG group for CGM to be cost neutral (total cost for both arms increased to £58,645,997, cost of consumables in the SMBG arm increases to £26,349,568).

### Scenario analysis

**Fig. 4:**
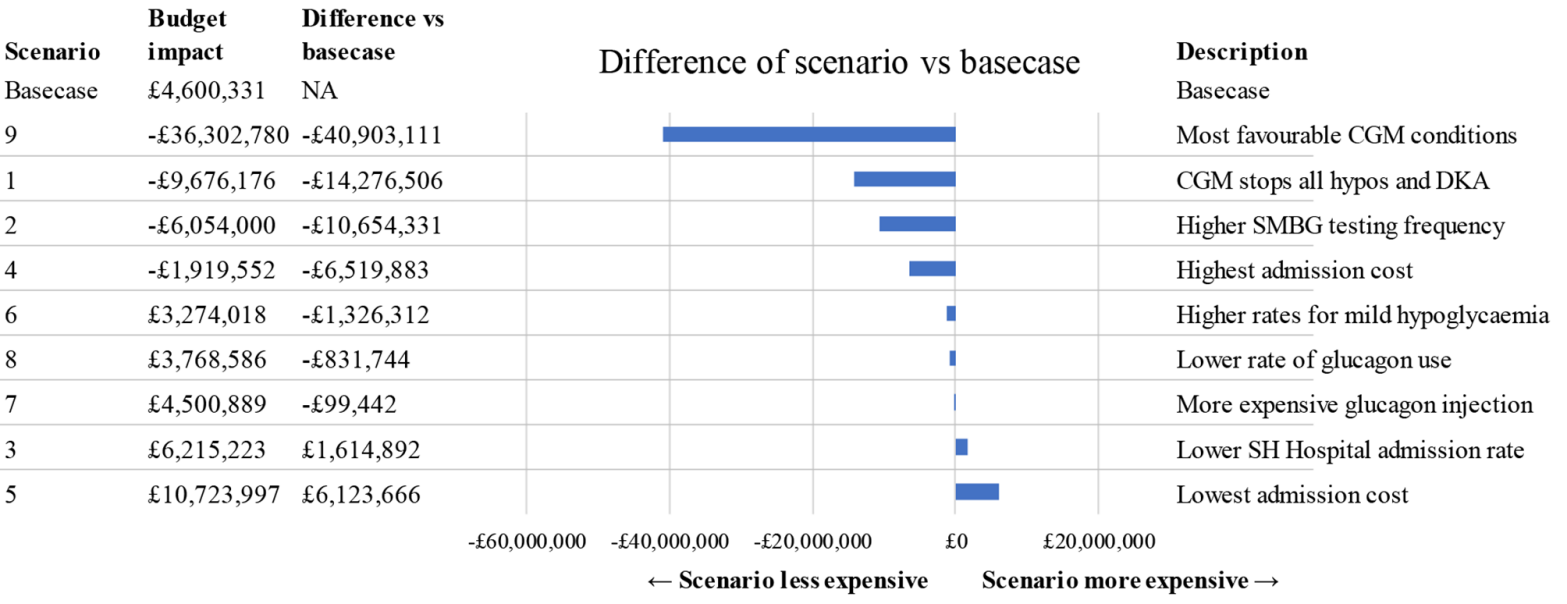
Scenario analysis. The “Budget impact” column is the budget of CGM vs. SMBG, where this is negative, CGM is cost saving vs. SMBG. CGM: Continuous glucose monitoring, DKA: Diabetic ketoacidosis, NA: Not applicable, SH: Severe hypoglycaemia, SMBG: Self-monitoring of blood glucose

Seven of nine scenarios were more favourable for CGM, compared with the basecase. In four scenarios, CGM was cost saving vs. SMBG, compared with SMBG: Scenario 1 (CGM stops all hypoglycaemia and DKA episodes), scenario 2 (higher SMBG testing frequency), scenario 4 (highest admission cost) and scenario 9 (most favourable CGM conditions). Scenario 3 (lower SH hospital admission rate) and scenario 5 (lowest SH and DKA admission cost) lead to a larger budget impact than the basecase. The scenario analysis results are presented in Fig. 4.

## Discussion

CGM has been shown to be clinically superior to SMBG at maintaining blood glucose levels within the euglycemic range [32]. Furthermore, a recent Swedish study showed long term improvements with CGM reducing HbA1c by 0.4% and it was cost-effective over the patient’s lifetime, both compared with SMBG [39].

Our England-based analysis has shown that CGM may result in increased spending in the NICE-eligible T2DM cohort in primary care, coupled with savings in secondary care, fewer hypoglycaemia and DKA events, and fewer attendances with almost all healthcare providers modelled. The BIA’s findings are validated by NICE’s own findings on CGM in T2DM [40] and are broadly in line with an England-based BIA in T1DM patients [12].

The cost increase of £4.6 million would be categorised by NICE as “Low cost” (cost increase between £1 million and £15 million per year). Technologies that lead to a cost increase of £15 million or more per year would be considered as “High cost” by NICE [41]. In addition, a budget impact test was introduced in England in 2017 [42], which triggers commercial negotiations with NHS England, if the projected budget impact of a new technology exceeds £20 million in any of the first three years of its use [43].

In a global context, our findings are consistent with analyses in other countries. For example, the Spain BIA in T2DM that uses basal-bolus insulin showed a potential cost-saving in this patient group [15], a finding that was echoed in the USA BIA of T2DM patients on intensive insulin treatment [16] and the Argentina BIA of T2DM patients on multiple daily insulin [17]. Our analysis showed a cost increase in T2DM on basal-only and premixed insulin, this was partially offset by a cost saving in T2DM basal-bolus and bolus only insulin users (Table 3).

The escalation to insulin treatment is critical to achieving HbA1c targets in some T2DM patients. This predisposes patients to hypoglycaemia, in turn requiring more frequent blood glucose testing [3], which may be painful and inconvenient with traditional tests and lancets [44, 45], not to mention the added cost of extra testing. However, given the opportunity, T2DM patients scanned the CGM device for their blood glucose levels very frequently in the pivotal study; 8.3 scans per day [32], and this was maintained in the 12 month extension at 7.1 times/day [20]. In real-world studies of people with unspecified diabetes, daily scanning was more frequent [46–48]. This suggests that testing with strips and lancets is associated with patient-level barriers that are resolved with the CGM device.

This BIA identified that 5.68 daily tests with traditional strips and lancets would lead to a neutral budget impact with CGM in the NICE-eligible population, aligning with the CGM eligibility criterion for those who must self-measure at least 8 times a day [3]. In these patients, a cost savings is anticipated. This agrees with the UK BIA in T1DM, which found CGM to be cost-saving in T1DM patients who test with traditional strips and lancets 10 times per day and cost-additive with 5.6 tests per day [11]. This may be considered external validation of our findings, resolving some modelling uncertainty.

This BIA estimates a 60.5% cost increase with CGM in primary care spending (from £27.2 million to £43.7 million), driven by CGM acquisition costs. However, £11.9 million is offset by savings in secondary care. Activity of GP practice nurses is anticipated to increase in the first year of roll-out, followed by a drop from the second year onwards as the number of new patients requiring CGM training is expected to decline.

In its totality, the SH treatment pathway modelled, builds on and goes beyond the costed elements in previous CGM BIAs [8–16]. Additionally, scenario 1 (CGM stops all hypoglycaemia and DKA events) was found to be cost saving. Considering newer models of the CGM device have alarms for high or low glucose levels, scenario 1 may be closer to what happens in current practice in England.

The DKA treatment pathway is likely to be similar to the SH treatment pathway. However, DKA is not as closely studied as hypoglycaemia, likely due to its lower incidence rate (e.g., SH in patients using basal-bolus insulin is 1.3 PPPY [49] versus 10.86 DKA episodes per 1,000 PPPY [50]; 100 x fold difference). Interestingly, this analysis estimates 5,682 SH and 2,880 DKA admissions in the NICE-eligible population; only a 2 times fold difference for those using SMBG. This implies that the proportion of DKA episodes that progress to an expensive hospital admission is substantially greater than SH. As such, greater efforts should be taken to ensure DKA episodes are resolved earlier, something the CGM device may contribute to.

The median incidence rates for mild hypoglycaemia reported by the UK Hypoglycaemia Study Group [22] used in the basecase seem to be heavily skewed by a subset of patients who experience more events than most insulin-treated patients. For example, for T2DM insulin users > 5 years, the median was 2.7 (range: 0 to 144) and the mean was 10.2 (95% CI: 5.4 to 15.0) [22]. As NICE criteria specify “recurrent / severe hypoglycaemia” [1], which could be interpreted as recurrent “mild” hypoglycaemia, the higher mean rate of 10.2 episodes PPPY used in Scenario 6 may better reflect the NICE eligible population, which is more favourable for CGM, compared with the basecase.

For the NICE-eligible population, the estimated number of glucose oral gel tubes prescribed (1,390,730) exceeds the total estimated mild hypoglycaemias (72,187) by 19 times. In the model, this led to the assumption that all mild hypoglycaemias are managed with prescribed oral glucose gel, which is highly unlikely. However, this approach was adopted to maintain the internal consistency of the model by using the same kind of data (prescribing data) to inform this kind of information (prescribed item usage). Medication waste and the lack of accurate data on hypoglycaemia are thought to be major contributors to this discrepancy. Future research here would be essential in reducing the uncertainty of this modelling input.

The inputs collated for this BIA were sourced from publicly available data. All inputs were critically appraised for suitability for this analysis to reduce modelling uncertainty. Some data and references were judged to be unsuitable. These are detailed in the Appendix.

### Limitations

Model inputs were gathered via a targeted literature search, however a systematic literature review would be considered the gold standard to ensure most accurate data with least uncertainty. The impact of CGM goes beyond the model’s perspective (direct costs to the commissioner); this model did not consider indirect cost savings or the wider societal impact. Our findings are further limited by our defined model scope, which could have omitted important cost drivers, model inputs were not validated by external decision-makers, as recommended by the ISPOR 2012 BIA Task Force [18], the rates used for hypoglycaemia and DKA may underestimate the actual rates in the NICE eligible T2DM population, the use of prescribing data to derive several model inputs and the lack of consideration for concomitant sulfonylurea use, which is known to cause or contribute to hypoglycaemia [5]. These and other limitations are fully detailed in the Appendix.

## Conclusions

Adopting CGM in the NICE-eligible T2DM population instead of SMBG may increase spending but reduce activity for most healthcare providers. CGM appears to be a long-term budget saving option for T1DM patients and has the potential to improve the clinical care of all CGM users.

Future research directions in T2DM could follow the same approach taken in a T1DM BIA, which included real-world data on HbA1c cost savings from a reduction in HbA1c [12], enabling a more accurate costing of CGM adoption in England in the NICE-eligible T2DM population.

### Electronic supplementary material

Below is the link to the electronic supplementary material.

Supplementary Material 1

## Data Availability

Not applicable.

## Abbreviations

A&E: Accident and Emergency
BIA: Budget impact analyses
CGM: Continuous glucose monitoring
DKA: Diabetic ketoacidosis
GP: General Practitioner
HbA1c: Glycated haemoglobin
HRG: Healthcare Resource Group
HES: Hospital Episode Statistics
PPPY: Per patient per year
SMBG: Self-monitoring of blood glucose
SH: Severe hypoglycaemia
T1DM: Type 1 diabetes mellitus
T2DM: Type 2 diabetes
