# Supplementary material for: Budget impact analysis of continuous glucose monitoring in individuals with type 2 diabetes on insulin treatment in England
Source: Health Econ Rev. 2024 May 6;14:32. doi: 10.1186/s13561-024-00505-7 (PMC11071237; doi:10.1186/s13561-024-00505-7)
Supplement: Supplementary file 1 — Supplementary Material 1 [file 13561_2024_505_MOESM1_ESM.docx]

# APPENDIX 1 (METHODOLOGY)

## Dividing the NICE-eligible population by insulin regimen

To split the NICE-eligible population, patient numbers from an England-based retrospective analysis were used to estimate the relative size of each subgroup; basal (462 patients), premixed (685 patients), basal-bolus (282 patients) and bolus only (27 patients) [1]. However, two types of insulins can be used as basal insulins; basal insulin analogues (once daily – outside of NICE recommendations) and basal human insulins (can be twice daily – potentially eligible) [2, 3]. The study [1] did not identify the type of basal insulin used, therefore, it was assumed that basal human insulin users were combined with basal insulin analogue users. Consequently, the basal insulin subgroup was further divided to remove patients who administer once daily from the modelled cohort by using prescribing data. Here, it was *assumed* that the volume of prescribed basal human insulin was representative of patients on basal human insulin. Primary care prescribing data in the year to June 2020 (to align with the ONS population used in the patient funnel) [4] for basal insulins listed in the BNF [2] are presented in Table s1 and Table s2.

**Table s1: Primary care prescribing of basal insulin analogues in England in the year to June 2020 [4]**

| **Insulin glargine 100 units/ml products** | **BNF code** | **Quantity** |
| --- | --- | --- |
| Cartridges (generic) | 0601012V0AAAAAA | 174,644 |
| Pre-filled devices (generic) | 0601012V0AAADAD | 381,087 |
| Cartridges (Lantus) | 0601012V0BBAAAA | 1,192,062 |
| Pre-filled devices (Lantus SoloStar) | 0601012V0BBAEAD | 5,633,883 |
| Cartridges (Abasaglar) | 0601012V0BDABAA | 80,846 |
| Pre-filled devices (Abasaglar KwikPen) | 0601012V0BDACAD | 922,525 |
| Pre-filled devices (Semglee) | 0601012V0BEAAAD | 49,762 |
| **Total** |  | **8,434,809** |
|  |  |  |
| **Insulin degludec 100 units/ml products** | **BNF code** | **Quantity** |
| Cartridges (generic) | 0601012Z0AAAAAA | 74,909 |
| Pre-filled devices (generic) | 0601012Z0AAABAB | 185,636 |
| Cartridges (Tresiba) | 0601012Z0BBAAAA | 327,341 |
| Pre-filled devices (Tresiba Flextouch) | 0601012Z0BBABAB | 1,403,786 |
| **Total** |  | **1,991,672** |
|  |  |  |
| **Insulin detemir 100 units/ml products** | **BNF code** | **Quantity** |
| Cartridges (generic) | 0601012X0AAAAAA | 103,307 |
| Pre-filled devices (generic) | 0601012X0AAABAB | 104,540 |
| Cartridges (Levemir) | 0601012X0BBAAAA | 1,414,859 |
| Pre-filled devices (Levemir FlexPen) | 0601012X0BBABAB | 2,710,718 |
| Pre-filled devices (Levemir InnoLet) | 0601012X0BBACAB | 51,111 |
| **Total** |  | **4,384,535** |

BNF: British National Formulary

**Table s2: Primary care prescribing of basal human insulins in England in the year to June 2020 [4]**

| **Insulin isophane human 100 units/ml products** | **BNF code** | **Quantity** |
| --- | --- | --- |
| Cartridges (generic) | 0601012S0AAALAL | 4,403 |
| Pre-filled devices (generic) | 0601012S0AAAIAI | 1,434 |
| Cartridges (Insulatard) | 0601012S0BDALAL | 745,260 |
| Pre-filled devices (Insulatard InnoLet) | 0601012S0BDAMAI | 368,441 |
| Cartridges (Humulin I) | 0601012S0BGADAL | 414,183 |
| Pre-filled devices (Humulin I KwikPen) | 0601012S0BGAFAI | 2,427,185 |
| Cartridges (Insuman Basal) | 0601012S0BIABAL | 10,377 |
| Pre-filled devices (Insuman Basal Solostar) | 0601012S0BIADAI | 220,413 |
| **Total** |  | **4,191,696** |

BNF: British National Formulary

Basal human insulins accounted for 22.1% of total basal insulins [4]. As such, the NICE-eligible basal insulin users were estimated as 102 patients.

The final generated percentages from these patient numbers were used to split the NICE eligible population into the four different insulin regimens. Please see Table s3 for final percentages used in the BIA.

**Table s3: Dividing the NICE-eligible population by insulin regimen**

| Insulin regimen | Patient numbers [1] | Adjustment [4]* | Final patient numbers | Percentages used in the BIA |
| --- | --- | --- | --- | --- |
| Basal | 462 | 22.1% | 102 | 9.3% |
| Premixed | 685 |  | 685 | 62.5% |
| Basal-bolus | 282 |  | 282 | 25.7% |
| Bolus | 27 |  | 27 | 2.5% |

*As described in Table s1 and Table s2. BIA: Budget impact analysis

Please note, basal human insulin administration instructions are once **or** twice a day [2]. No publications were found that report on the number of patients who specifically administer twice daily. The analysis *assumes* that all basal human insulin users use the treatment twice a day. Including all basal human insulin users, would increase the size of this group, relative to the other three groups; over-representing their cost impact as part of the total cost. This was thought to generate avoidable uncertainty.

## Estimating the size of different insulin user populations in England

This model utilises prescribing data to estimate the approximate usage of glucose oral gel and glucagon injections in the NICE-eligible population. However, prescribing data are not specific to an indication or patient group. Therefore, the model assumes these medicines are used evenly in all relevant patient groups, including the NICE-eligible population of T2DM patients.

The model starts by estimating the number of insulin users in England:

- T2DM insulin users
  - NICE-eligible population: 48,797
  - Multiple daily insulin injectors who do not have impaired awareness, recurrent hypoglycaemia, SH and do not test ≥8 times/day (not NICE-eligible): 56,596
  - Those who inject once daily (not NICE-eligible): 34,630
- T1DM (assumed all use insulin):
  - Adults: 263,896
  - Under 18's: 27,646

This segmentation was adopted to align with different rates of mild or severe hypoglycaemia and the NICE patient funnel [5]. The method of derivation for these figures are detailed below.

- - 1. ***The number of T2DM patients who use insulin in England***

The number of T2DM multiple daily insulin injectors and the subset who are eligible, as per NICE, were previously described in section ‎1.1. By subtracting those who are eligible (48,797) from the total T2DM multiple daily insulin injectors (105,393), the T2DM multiple daily insulin injectors who are not NICE-eligible are calculated as 56,596 patients.

The size of the T2DM who inject once daily can be estimated with the relative proportion of this group to all T2DM patients who use insulin. Based on the segmentation of the T2DM population who use insulin (described in section ‎1.1), we can present the population previously described [1] as:

- T2DM patients who inject once daily (with basal insulin analogues): 360 patients (24.7%)
- T2DM multiple daily insulin injectors (with basal human insulins, premixed insulins, basal-bolus and bolus only): 1,096 patients (75.3%).

According to the NICE patient funnel, there are an estimated 105,393 patients with T2DM who inject insulin multiple times per day in England (Please see Results in the main paper). This patient group represents 75.3% of all T2DM patients who use insulin, as calculated above. Therefore, the remaining 24.7% of patients who inject insulin once daily can be estimated as 34,630 T2DM patients.

- - 1. ***The number of T1DM patients in England***

It is assumed all T1DM patients inject insulin.

In England, the NICE patient funnel estimates 263,896 adults with T1DM and 27,646 under 18's with T1DM. This is detailed in Table s4.

**Table s4: The number of T1DM patients in England**

| **Adults with T1DM** | **Proportion** | **Number** | **Reference** |
| --- | --- | --- | --- |
| England population in 2020 | - | 56,550,138 | [6] |
| Adults (18 and over) | 78.6% | 44,456,850 | [6] |
| Prevalence of diabetes mellitus | 7.4% | 3,298,698 | [5] |
| Proportion that are T1DM | 8% | 263,896 | [5] |
|  |  |  |  |
| **Under** **18's with T1DM** | **Proportion** | **Number** | **Reference** |
| England population in 2020 | - | 56,550,138 | [6] |
| Under 18's | 21.4% | 12,093,288 | [5] |
| Prevalence of diabetes mellitus in under 18's | 0.25% | 30,718 | [5] |
| Proportion that are T1DM | 90% | 27,646 | [5] |

T1DM: Type 1 diabetes mellitus

- - 1. ***Proportion of mild hypoglycaemia, which utilised oral glucose gel***

Glucose 40% oral gel (75 g) is reimbursed by the National Health Service (NHS) in England [7]. To estimate the proportion of mild hypoglycaemias, which utilised Glucose 40% oral gel, prescribing data for all 75 g glucose 40% oral gel products listed in the BNF [2] were extracted [4] in the year July 2019 to June 2020 (to align with the ONS population used in the patient funnel). According to the prescribing data, there were 28,792,495 packs of oral glucose 75 g prescribed in England [4], detailed in Table s5 [4].

**Table s5: Primary care prescribing of glucose 40% oral gel 75 gram in the year to June 2020** [4]

| **Glucose 40% oral gel 75 gram products** | **BNF code** | **Quantity** |
| --- | --- | --- |
| Generic | 0601040I0AAADAD | 13,533,676 |
| Dextrogel | 0601040I0BEAAAD | 1,591,661 |
| GlucoBoost | 0601040I0BGAAAD | 120,346 |
| GlucoGel berry | 0601040I0BBAEAD | 20,200 |
| GlucoGel original | 0601040I0BBABAD | 12,116,687 |
| Rapilose | 0601040I0BFAAAD | 1,152,600 |
| YourGLUCO | 0601040I0BHAAAD | 257,325 |
| **Total** |  | **28,792,495** |

BNF: British National Formulary

Each 75 g pack contains 3 tubes of 25 g and the model assumes 1 tube is used to treat 1 mild hypoglycaemia episode. Therefore, it is assumed that glucose 40% oral gel was used to treat 86,377,485 mild hypoglycaemia episodes in 1 year.

The prescribing data do not distinguish between indications and different patient groups. To estimate how many tubes were used in the NICE-eligible population, the total number of mild hypoglycaemia episodes in England was estimated. The reported median incidence of mild self-reported hypoglycaemia in T2DM insulin users and T1DM patients is summarised in Table s6[8].

**Table s6: Self-reported incidence of mild self-reported hypoglycaemia in T1DM and T2DM insulin users** [8]

|  | **n** | **Median PPPY (range)** |
| --- | --- | --- |
| T2DM insulin users <2 years | 85 | 1 (0 to 44) |
| T2DM insulin users >5 years | 75 | 2.7 (0 to 144) |
| T2DM weighted average* |  | 1.80 |
| T1DM <5 years | 46 | 22.3 (0 to 203) |
| T1DM >15 years | 54 | 13.7 (0 to 290) |

*Calculated, not reported in the publication

PPPY: Per patient per year, T1DM: Type 1 diabetes mellitus, T2DM: Type 2 diabetes mellitus

By multiplying these rates by the number of patients calculated in section ‎1.2, the total mild hypoglycaemias in England per year are estimated as:

- T2DM multiple daily insulin injectors: 189,379 mild hypoglycaemia episodes (using the weighted average rate for T2DM)
- T2DM who inject once daily 62,225 episodes (using the weighted average rate for T2DM)
- Adults with T1DM: 3,615,373 episodes (using the rate for T1DM >15 years)
- Under 18's with T1DM: 616,516 episodes (using the rate for T1DM <5 years)

These sum to 4,483,494 mild hypoglycaemias in England per year. The NICE-eligible cohort experience an estimated 72,187 mild hypoglycaemias or 1.61% of all mild hypoglycaemias.

By applying this percentage to 86,377,485 tubes of 25 g glucose oral gel, we can estimate that 1,390,730 tubes of 25 g glucose oral gel were used in the NICE-eligible population.

From here, usage would have been estimated by dividing the number of glucose oral gel (1,390,730) by the total mild hypoglycaemias (72,187) in the NICE-eligible population. However, the prescribed glucose oral gel exceeds mild hypoglycaemias by 19 times.

This model aims to estimate the cost associated with mild hypoglycaemia in T2DM insulin users, which includes prescribed glucose oral gel. As prescribing exceeds estimated mild hypoglycaemias, the model will assume 100% of mild hypoglycaemias are treated with glucose oral gel. Some of this will be medication waste, which is included in the model, as it is incurred by commissioners. Prescribing in excess of mild hypoglycaemias is considered to be out of scope of this analysis, even though it is a substantial volume.

- - 1. ***Annual rates of SH***

The incidence of basal insulin used was 0.173 PPPY, which was reported in a systematic review of 76 observational (retrospective or prospective) studies encompassing 707,722.30 patient-years [9].

The incidence of premixed insulin was 0.168 PPPY, based on the weighted average for premixed insulin analogues (0.092 PPPY [9]) and premixed human insulin (0.299 PPPY [9]) weighted by the volume prescribed in the year to June 2020 [4] showed that 63.2% of prescribing was for premixed insulin analogues and the remaining 36.8% were for premixed human insulins. Primary care prescribing data for premixed insulins listed in the BNF [2] are presented in Table s7 and Table s8 below.

**Table s7: Primary care prescribing of premixed insulin analogues in England in the year to June 2020 [4]**

| **Insulin aspart biphasic 30/70 100 units/ml products** | **BNF code** | **Quantity** |
| --- | --- | --- |
| Cartridges (generic) | 0601012W0AAAAAA | 36 |
| Pre-filled devices (generic) | 0601012W0AAABAB | 1,292 |
| Cartridges (Novomix) | 0601012W0BBAAAA | 1,372,655 |
| Pre-filled devices (Novomix Flexpen) | 0601012W0BBABAB | 5,598,406 |
| **Total** |  | **6,972,389** |
|  |  |  |
| **Insulin lispro biphasic 100 units/ml products** | **BNF code** | **Quantity** |
| 25/75 Cartridges (generic) | 0601012F0AAABAB | 795 |
| 25/75 Pre-filled devices (generic) | 0601012F0AAAAAA | 45 |
| 50/50 Cartridges (generic) | 0601012F0AAADAD | 30 |
| 50/50 Pre-filled devices (generic) | 0601012F0AAACAC | 120 |
| 25/75 Cartridges (Humalog Mix25) | 0601012F0BBABAB | 526,210 |
| 25/75 Pre-filled devices (Humalog Mix25 KwikPen) | 0601012F0BBAEAA | 1,036,854 |
| 50/50 Cartridges (Humalog Mix50) | 0601012F0BBADAD | 250,865 |
| 50/50 Pre-filled devices (Humalog Mix50 KwikPen) | 0601012F0BBAFAC | 1,317,203 |
| **Total** |  | **3,132,122** |

BNF: British National Formulary

**Table s8: Primary care prescribing of premixed human insulins in England in the year to June 2020** [4]

| **Insulin isophane biphasic 100 units/ml products** | **BNF code** | **Quantity** |
| --- | --- | --- |
| 25/75 Cartridges (generic) | 0601012D0AABNBN | 20 |
| 25/75 Pre-filled devices (generic) | 0601012D0AABWBW | 81 |
| 30/70 Cartridges (generic) | 0601012D0AABABA | 275 |
| 30/70 Pre-filled devices (generic) | 0601012D0AABZBZ | 293 |
| 50/50 Cartridges (generic) | 0601012D0AABUBU | 32 |
| 25/75 Cartridges (Insuman Comb) | 0601012D0BGABBN | 15,134 |
| 25/75 Pre-filled devices (Insuman Comb 25 SoloStar) | 0601012D0BGAJBW | 175,522 |
| 30/70 Cartridges (Humulin M3) | 0601012D0BBASBA | 1,274,088 |
| 30/70 Pre-filled devices (Humulin M3 KwikPen) | 0601012D0BBAVBZ | 4,378,862 |
| 50/50 Cartridges (Insuman Comb 50) | 0601012D0BGAFBU | 48,647 |
| **Total** |  | **5,892,954** |

BNF: British National Formulary

In line with the T2DM BIA of CGM in Spain, the incidence for patients on basal-bolus insulin used was 2.5 PPPY [10]. The rate for bolus insulin users was assumed to be the same; 2.5 PPPY.

- - - 1. ***Step 1: glucagon injection at home***

No publications were found that presented an estimate of SH that were treated with prescribed glucagon 1 mg injection. Therefore, the same calculation approach as glucose oral gel usage in mild hypoglycaemia was used to estimate this rate; dividing the prescribed glucagon 1 mg injection by total SH episodes, both in the NICE-eligible population.

The total number of SH was calculated as 40,314 per year. The total prescribed volume of glucagon 1 mg injections in England in the year to June 2020 were 99,609 vials [4]. Primary care prescribing data in the year to June 2020 (to align with the ONS population used in the patient funnel) [4] for glucagon 1 mg injections listed in the BNF [2] are presented in Table s9.

**Table s9: Primary care prescribing of glucagon 1 mg injections in the year July 2019 to June 2020 [4]**

| **Glucagon 1 mg injection products** | **BNF code** | **Quantity** |
| --- | --- | --- |
| Vials (generic) | 0601040H0AAAEAE | 41,268 |
| Vials (GlucaGen Hypokit) | 0601040H0BDAAAE | 58,341 |
| Pre-filled devices (generic) | 0601040H0AAAFAF | 0 |
| Pre-filled devices (Ogluo) | 0601040H0BEAAAF | 0 |
| **Total** |  | **99,609** |

BNF: British National Formulary

To estimate the proportion that was used in the NICE-eligible population, it was assumed that the proportion of the NICE-eligible SH of the total SH for all patients was representative of the portion of glucagon 1 mg injection used in the NICE-eligible population; 3.6%. Therefore, the model assumes 3,602 glucagon 1 mg injections were used in 40,314 SH episodes; i.e., 8.9% of SH episodes. Full details of the calculations and references are presented in Table s10.

**Table s10: Method of estimating the proportion of severe hypoglycaemia episodes where glucagon 1 mg injections were used**

|  |  | **Number** | **Notes** | **Reference** |
| --- | --- | --- | --- | --- |
| A | NICE-eligible population | 48,797 | See Results in the main paper for calculation method |  |
| B | T2DM patients who use insulin | 140,023 | See section ‎1.2.1 for calculation method |  |
| C | T1DM patients | 291,542 | See section ‎1.2.2 for calculation method |  |
| D | NICE-eligible population SH PPPY | Several | See Section ‎1.2.4 for details |  |
| E | T2DM SH PPPY | 1.3 | The rate of SH in T2DM insulin users in Northern Europe/Canada | [11] |
| F | T1DM SH PPPY | 3.2 | The rate of SH in T1DM used in the CGM BIA for T1DM patients in the UK | [12] |
| G | NICE-eligible population SH annual episodes | 40,314 | Number of patients multiplied by SH PPPY | Calculated: row A x row D |
| H | T2DM SH annual episodes | 182,030 | Number of patients multiplied by SH PPPY | Calculated: row B x row E |
| I | T1DM SH annual episodes | 932,935 | Number of patients multiplied by SH PPPY | Calculated: row C x row F |
| J | NICE-eligible population SH annual episodes % | 3.6% | NICE-eligible population SH divided by all SH | Calculated: row G ÷ [rows H + I] |
| K | Prescribed glucagon 1 mg injections in England | 99,609 | See Table s9 for details | [4] |
| L | Proportion of T2DM SH that use glucagon 1 mg injections | 3,602 | Multiply the NICE-eligible population SH annual episodes % by the number of prescribed glucagon 1 mg injections in England | Calculated: row J x row K |
| **M** | **% NICE-eligible population SH that use glucagon 1 mg injections** | **8.9%** | Divide proportion of SH that use glucagon by SH episodes/year, both in the NICE-eligible population | Calculated: row L ÷ row G |

PPPY: per patient per year, SH: severe hypoglycaemia, T1DM: Type 1 diabetes mellitus, T2DM: Type 2 diabetes mellitus

- - - 1. ***Step 2: Ambulance emergency treatment***

If the patient does not respond normally or is unconscious, and a glucagon injection is not available, a glucagon injection is not effective within 10 minutes or if they have been drinking alcohol, the NHS advises contacting emergency services [13], starting with an ambulance.

Ambulance teams were modelled to resolve SH in 90.1% of cases. This was calculated from a retrospective analysis of data collected by the East Midlands Ambulance Trust in England [14] over the period 01/11/10-28/02/2011 [15]. In their analysis, the authors found that 32% of SH cases were transported to hospital [14] and 68% would have been resolved by ambulance teams. However, not all patients were treated with insulin in this study. Insulin treatment significantly reduced the likelihood of hospitalisation based on multivariate regression analyses (odds ratio 0.31, 95% confidence interval 0.15 to 0.64, p<0.01) [15].

Therefore, the SH rate of resolution was calculated by:
100% - (32% x 0.31) = 90.1%

- - - 1. ***Step 4: Hospital admission***

In Step 4, patients are admitted to hospital, at a cost of £2,461. According to the 2023/24 Prices Workbook, non-elective hypoglycaemia spells cost between £570 to £3,901 [16]. The cost increases as the complexity and comorbidity (CC) score rises and whether the spell was a long or short stay. The cost of admission was the average guide price (non-elective and adjusted non-elective for reduced short stay emergency admissions), weighted by the activity (long stay and short stay). Please see Table s11 for guide prices [16] and activity [17] for each type.

**Table s11: Non-elective inpatient care for admitted patients with hypoglycaemia**

| **Long stay** | | | |
| --- | --- | --- | --- |
| **HRG Code** | **HRG Name** | **Guide price (£)** [16] | **Long stay activity** [17] |
| KB01C | Diabetes with Hypoglycaemic Disorders, with CC Score 8+ | £3,901 | 5,296 |
| KB01D | Diabetes with Hypoglycaemic Disorders, with CC Score 5-7 | £1,901 | 1,679 |
| KB01E | Diabetes with Hypoglycaemic Disorders, with CC Score 3-4 | £1,132 | 692 |
| KB01F | Diabetes with Hypoglycaemic Disorders, with CC Score 0-2 | £570 | 228 |
|  |  |  |  |
| **Short stay** | | | |
| **HRG Code** | **HRG Name** | **Adjusted short stay emergency price (£)*** [16] | **Short stay activity** [17] |
| KB01C | Diabetes with Hypoglycaemic Disorders, with CC Score 8+ | £3,121 | 3,589 |
| KB01D | Diabetes with Hypoglycaemic Disorders, with CC Score 5-7 | £1,046 | 2,253 |
| KB01E | Diabetes with Hypoglycaemic Disorders, with CC Score 3-4 | £396 | 1,407 |
| KB01F | Diabetes with Hypoglycaemic Disorders, with CC Score 0-2 | £570 | 851 |
|  |  |  |  |
| **Weighted average (short and long stays)** | | **£2,461** |  |

*The adjusted price was calculated by deducting the "Reduced short stay emergency adjustment (£)" from the main guide price. Admissions with CC Score 0-2 had no adjustment price.

CC: Complexity and comorbidity, HRG: Healthcare Resource Group

In the management of hypoglycaemia admissions, UK guidelines recommend a review “by a specialist diabetes physician or nurse before discharge” [18]. A previously published analysis of hypoglycaemia costs in the UK added the cost of a specialist to the hospital attendance cost [19]. However, to avoid duplication of costs, it is assumed that the cost of this review is included in the guide price described above.

## Diabetic ketoacidosis (DKA)

- - 1. ***Annual rates***

To estimate how many DKA admissions related to the NICE-eligible T2DM population, the incidence of insulin usage compared with non-insulin usage was used to exclude non-insulin-related DKA episodes. Then, the proportion of the NICE-eligible population of the whole T2DM insulin using population was used to funnel down to the total DKA admissions, which could be related to the NICE-eligible population. This is explained below.

According to a previous analysis of hospital admission for DKA in Diabetes in England, the incidence of DKA in T2DM patients varied based on their treatment mix; insulin use showed a marked increase in incidence, compared to patients not taking insulin [20]. The incidence rates are presented in Table s12.

**Table s12: Incidence of hospital admission for DKA in T2DM in 2013 by type of treatment**

|  | **Episodes per 1,000 PPPY** [21] | **Calculated percentage of episodes** |
| --- | --- | --- |
| Sulfonylureas with or without NIGLD | 0.44 | 2.9% |
| Insulin only | 10.86 | 71.7% |
| Insulin with NIGLD | 3.71 | 24.5% |
| Other | 0.14 | 0.9% |

NIGLD: non-insulin glucose-lowering drugs, Other: self-management only and monotherapy or combination of non-insulin glucose-lowering drugs (excluding sulfonylureas), PPPY: Per patient per year

As can be seen from Table s12, 96.2% of DKA admissions are in patients who are treated with insulin. By applying this rate to the HES number of admissions (8,592 admissions) [22], we can calculate the number of admissions related to T2DM patients on insulin:

8,592 x 96.2% = 8,263 DKA admissions in all T2DM on insulin

As detailed in section ‎1.2.1, there are an estimated 140,023 T2DM patients who inject insulin in England. Of those, the NICE-eligible population (48,797 patients) represents 34.8%. By applying this percentage to the number of DKA episodes above:

8,263 x 34.8% = 2,880 DKA admissions in the NICE-eligible population.

This crude method of estimating DKA incidence yields a rate of 0.059 per NICE-eligible patient in 1 year.

- - 1. ***Unit costs per DKA admission***

The cost of a DKA admission (£1,938) was derived using the same method as an SH admission; the average guide price [16] of Diabetes with Hyperglycaemic Disorders, weighted by their respective activities reported in NHS National Cost Collection data [17]. As above, prices and activities of both non-elective and reduced short stay emergency admissions were used. Details of costs and activities are summarised in Table s13.

**Table s13: Non-elective inpatient care for admitted patients with DKA**

| **Long stay** | | | |
| --- | --- | --- | --- |
| **HRG Code** | **HRG Name** | **Guide price (£)** [16] | **Long stay activity** [17] |
| KB02G | Diabetes with Hyperglycaemic Disorders, with CC Score 8+ | £4,138 | 10,487 |
| KB02H | Diabetes with Hyperglycaemic Disorders, with CC Score 5-7 | £2,259 | 6,089 |
| KB02J | Diabetes with Hyperglycaemic Disorders, with CC Score 2-4 | £1,389 | 6,247 |
| KB02K | Diabetes with Hyperglycaemic Disorders, with CC Score 0-1 | £934 | 2,587 |
|  |  |  |  |
| **Short stay** | | | |
| **HRG Code** | **HRG Name** | **Adjusted short stay emergency price* (£)** [16] | **Short stay activity** [17] |
| KB02G | Diabetes with Hyperglycaemic Disorders, with CC Score 8+ | £3,310 | 6,302 |
| KB02H | Diabetes with Hyperglycaemic Disorders, with CC Score 5-7 | £1,242 | 6,327 |
| KB02J | Diabetes with Hyperglycaemic Disorders, with CC Score 2-4 | £486 | 10,424 |
| KB02K | Diabetes with Hyperglycaemic Disorders, with CC Score 0-1 | £934 | 8,090 |
|  |  |  |  |
| **Weighted average (short and long stays)** | | **£1,938** |  |

*The adjusted price was calculated by deducting the "Reduced short stay emergency adjustment (£)" from the main guide price. Admissions with CC Score 0-2 had no adjustment price.

CC: Complexity and comorbidity, HRG: Healthcare Resource Group

The involvement of specialists is repeatedly recommended in the management of DKA admissions by UK guidelines [23]. As for SH, it is assumed that the cost of specialist team involvement is included in the guide price described above, to avoid duplication of costs.

- 1. ***Consumables***

### ***Deriving the price of ketone testing***

The price was derived by calculating the weighted average price of all tests listed in the BNF[2], weighted by the volume prescribed in the year to June 2020, inclusive[4]. For blood test products, £0.04 was added to the cost of each test/strip to account for the price of a lancet [12].

**Table s14: Primary care prescribing of blood and urine ketone testing strips in England in the year to June 2020** [2, 4]

| **Ketone testing strips** | **BNF code** | **Pack size** [4] | **Price per pack** [4] | **Quantity** [4] |
| --- | --- | --- | --- | --- |
| GlucoRx KetoRx Sticks 2GK testing strips (urine) | 0601060U0BQAAA0 | 50 | £2.25 | 65,250 |
| Ketostix testing strips (urine) | 0601060U0BHAAA0 | 50 | £3.11 | 3,191,753 |
| 4SURE beta-ketone testing strips | 0601060W0BGAAA0 | 10 | £9.92 | 60,371 |
| Fora Advanced pro GD40 Ketone testing strips | 0601060W0BHAAA0 | 10 | £8.95 | 19,180 |
| FreeStyle Optium beta-ketone testing strips | 0601060W0BCAAA0 | 10 | £21.94 | 2,998,616 |
| GlucoMen areo Ketone Sensor testing strips | 0601060W0BDABA0 | 10 | £9.95 | 1,221,210 |
| GlucoRx HCT Ketone testing strips | 0601060W0BEAAA0 | 10 | £9.95 | 282,900 |
| GlucoRx X6 Ketone testing strips | 0601060W0BJAAA0 | 10 | £15.95 | 0 |
| KetoSens testing strips | 0601060W0BFAAA0 | 10 | £9.95 | 449,500 |
| **Weighted average price per test*** | **£1.09** |  |  |  |

Urine tests are indicated with “urine” in brackets.

*For ketone blood test products, £0.04 was added to the cost of each test/strip to account for the price of a lancet [12].

BNF: British National Formulary

# APPENDIX 2 (RESULTS)

## Detailed Basecase output

**Table s15: Detailed Basecase output for the overall activity outcomes, and primary vs secondary care split**

| **Overall SMBG activity** | **Basal only** | **Premixed** | **Basal-bolus** | **Bolus only** | **Total** |
| --- | --- | --- | --- | --- | --- |
| SH | 847 | 5,539 | 33,880 | 3,244 | 43,509 |
| DKA | 399 | 2,681 | 1,104 | 106 | 4,290 |
| GP practice | 7,809 | 52,203 | 85,534 | 8,189 | 153,736 |
| Total | 9,056 | 60,423 | 120,517 | 11,539 | 201,535 |
|  |  |  |  |  |  |
| **SMBG SH activity** | **Basal only** | **Premixed** | **Basal-bolus** | **Bolus only** | **Total** |
| Ambulance | 210 | 1,371 | 8,387 | 803 | 10,770 |
| Hospital A&E | 527 | 3,444 | 21,069 | 2,017 | 27,057 |
| Hospital admission | 111 | 723 | 4,424 | 424 | 5,682 |
| Total | 847 | 5,539 | 33,880 | 3,244 | 43,509 |
|  |  |  |  |  |  |
| **SMBG DKA activity** | **Basal only** | **Premixed** | **Basal-bolus** | **Bolus only** | **Total** |
| Hospital A&E | 131 | 881 | 363 | 35 | 1,410 |
| Hospital admission | 268 | 1,800 | 741 | 71 | 2,880 |
| Total | 399 | 2,681 | 1,104 | 106 | 4,290 |
|  |  |  |  |  |  |
| **SMBG GP practice activity** | **Basal only** | **Premixed** | **Basal-bolus** | **Bolus only** | **Total** |
| GP | 6,757 | 45,271 | 53,402 | 5,113 | 110,542 |
| Practice Nurse | 1,053 | 6,932 | 32,132 | 3,076 | 43,193 |
| Total | 7,809 | 52,203 | 85,534 | 8,189 | 153,736 |
|  |  |  |  |  |  |
| **Overall CGM activity** | **Basal only** | **Premixed** | **Basal-bolus** | **Bolus only** | **Total** |
| SH | 467 | 3,051 | 18,664 | 1,787 | 23,969 |
| DKA | 220 | 1,477 | 608 | 58 | 2,363 |
| GP practice | 10,878 | 72,957 | 65,316 | 6,254 | 155,405 |
| Total | 11,564 | 77,485 | 84,588 | 8,099 | 181,737 |
|  |  |  |  |  |  |
| **CGM SH activity** | **Basal only** | **Premixed** | **Basal-bolus** | **Bolus only** | **Total** |
| Ambulance | 116 | 755 | 4,620 | 442 | 5,933 |
| Hospital A&E | 290 | 1,897 | 11,607 | 1,111 | 14,906 |
| Hospital admission | 61 | 398 | 2,437 | 233 | 3,130 |
| Total | 467 | 3,051 | 18,664 | 1,787 | 23,969 |
|  |  |  |  |  |  |
| **CGM DKA activity** | **Basal only** | **Premixed** | **Basal-bolus** | **Bolus only** | **Total** |
| Hospital A&E | 72 | 486 | 200 | 19 | 777 |
| Hospital admission | 148 | 992 | 408 | 39 | 1,586 |
| Total | 220 | 1,477 | 608 | 58 | 2,363 |
|  |  |  |  |  |  |
| **CGM GP practice activity** | **Basal only** | **Premixed** | **Basal-bolus** | **Bolus only** | **Total** |
| GP | 5,760 | 38,638 | 35,058 | 3,357 | 82,812 |
| Practice Nurse | 5,118 | 34,319 | 30,258 | 2,897 | 72,592 |
| Total | 10,878 | 72,957 | 65,316 | 6,254 | 155,405 |

A&E: accident and emergency, CGM: Continuous glucose monitoring, DKA: diabetic ketoacidosis, GP: general practitioner, SH: Severe hypoglycaemia, SMBG: Self-monitoring of blood glucose

**Figure s1: Budget impact on primary vs secondary care**

Items included in primary care: Mild hypoglycaemia, SH – Glucagon, consumables, GP practice costs. Items included in secondary care: SH – Ambulance, SH ‑ Hospital A&E, SH - Hospital admission, DKA ‑ Hospital A&E, DKA - Hospital admission.

CGM: Continuous glucose monitoring, DKA: diabetic ketoacidosis, GP: general practitioner, SH: Severe hypoglycaemia, SMBG: Self-monitoring of blood glucose

**Figure s2: SH in SMBG: costs vs activity comparison**

A&E: accident and emergency, SH: Severe hypoglycaemia, SMBG: Self-monitoring of blood glucose

**Figure s3: GP practice comparison**

| **Cost** | **Activity** |
| --- | --- |
|  |  |
| GP Practice nurse | |
| CGM: Continuous glucose monitoring, GP: general practitioner, SMBG: Self-monitoring of blood glucose | |

## Budget and capacity impact in an average ICS and for a population of 1,000,000

The budget impact for an average ICS was increased spending by £109,532 but activity was reduced by 471 attendances in primary and secondary care.

**Figure s4: Overall budget and activity impact for an average ICS and a population of 1,000,000**

| **Average ICS (1,346,432 population)** | |
| --- | --- |
| **Budget impact: £109,532 increase in costs**   | **Activity impact: 471 avoided attendances**   |
| **Population of 1,000,000** | |
| **Budget impact: £81,350 increase in costs**   | **Activity impact: 350 avoided attendances**   |

CGM: Continuous glucose monitoring, DKA: diabetic ketoacidosis, GP: general practitioner, hypos: hypoglycaemias, SH: Severe hypoglycaemia, SMBG: Self-monitoring of blood glucose.

# APPENDIX 3 (UNUSED INPUTS)

## Why figures from the UK-based T1DM BIA were not included

The UK-based T1DM BIA includes admission cost in one scenario; 11.8% of SH episodes accrue £1,133.78 per episode for a hospital admission [12]. The rate of 11.8% references a review of US studies on the economic impact of diabetes [24]. The way the BIA utilises their input may not agree with their reference as the rate of 11.8% was the proportion of SH episodes that required medical assistance [12, 24], however, they cost all medical assistance at the rate of hospital admission, which is the highest possible cost in the SH treatment pathway. If the data published by Foos, et al. [24] is to be used appropriately, some patients would be treated by Ambulance or hospital A&E. Only a small proportion would be admitted to hospital [25]. None of this is considered in the T1DM BIA [12].

## Why figures from an England-based SH emergency treatment, peer-reviewed publication were not used

In an England-based study, 35.3% of SH patients were taken to hospital by ambulance in the year to November 2010 [26]. In this peer-reviewed publication, it was assumed that the presence of hypoglycaemia is causally related to insulin [26]. However, no data were presented to inform which patients were treated with insulin and subsequently transported to hospital A&E. This is relevant, as insulin treatment significantly reduced the likelihood of hospitalisation after treatment by ambulance teams (p<0.01) [15]. Furthermore, the split of SH occurring in T1DM vs T2DM was not presented [26].

Therefore, these data were not used.

## Why SH A&E and admission model inputs did not utilise HES data

The SH treatment pathway did not use annual published HES data for England as hypoglycaemia episodes could be coded using several codes. Furthermore, the episode label does not always distinguish between T1DM or T2DM. To use HES data for hypoglycaemia-related inputs, several assumptions would need to be made to infer which proportion of those admissions may relate to the NICE-eligible population. This was thought to introduce more uncertainty compared with the approach adopted.

## Why a previous peer-reviewed publication on DKA incidence was not used

A previous HES analysis of hospital admission for DKA in diabetes in England [20] showed the incidence of DKA in T2DM using insulin only was 0.011 PPPY, while those on insulin and oral antidiabetics experienced 0.0037 DKA episodes PPPY [21].

These data were not used in the basecase as the publication showed a dramatic rise in DKA incidence between 1997 and 2013 for patients on insulin, as can be seen in Figure s5.

**Figure s5: Trends in hospital admission for diabetic ketoacidosis in T2DM adults, by age use of glucose-lowering drugs** [21]


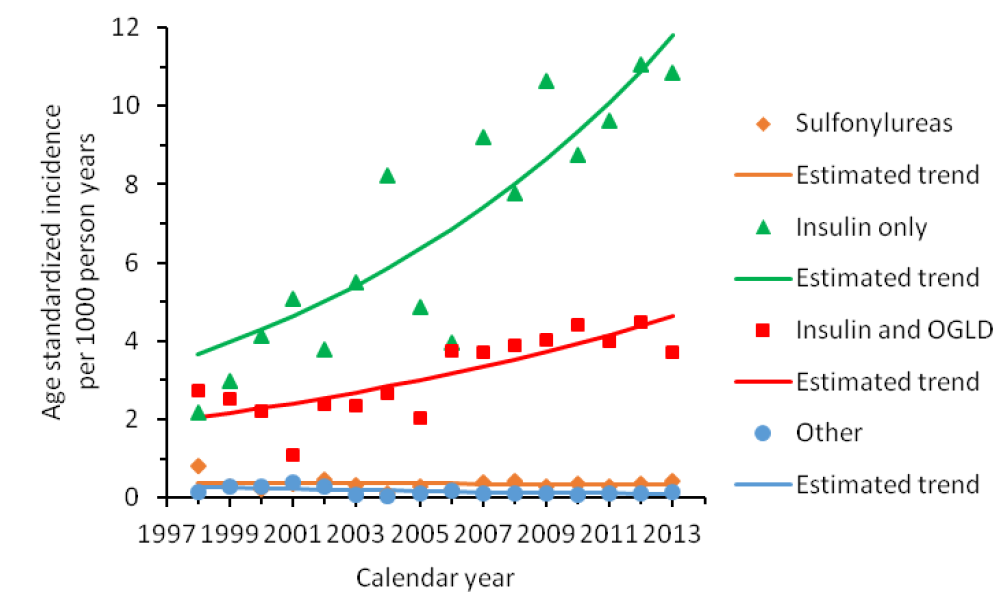


OGLD: oral glucose-lowering drug

The data in this analysis goes up to 2013 and the curve shows no signs of levelling out. To avoid underestimating DKA events, current HES data were used. The crude DKA incidence rate calculated in this BIA for the NICE-eligible population was 0.059 PPPY, 5 times higher than the reported rate for insulin-only users and 15 times higher than those using insulin and oral antidiabetics, potentially validating the approach adopted.

# APPENDIX 4 (LIMITATIONS)

## Limitations of the literature search

A targeted literature search was performed for model inputs. However, a 2015 publication “highlighted the lack of data on hypoglycaemia rates and resource use for the treatment of severe and non-severe episodes” [19]. Hypoglycaemia resource utilisation is a major cost component of this model. A systematic literature review is the gold standard for ensuring the best data are used to develop the model with minimal uncertainty.

## Items considered out of scope of the BIA

This model does not include all items that may be impacted by the introduction of CGM. To start, it only considers direct costs to the payer, the model does not consider the wider societal impact. Hypoglycaemia has been “associated with substantial economic consequences for employers and patients” [27] and “poor health outcomes, such as reduced health‐related quality of life” [28]. SH lead to a greater impact on productivity and quality of life, compared with mild episodes [28].

The NICE committee considered the impact of CGM to be minimal on HbA1c [3], in line with the pivotal study, which showed no change with CGM in the HbA1c of 149 participants after 6 months [29]. To manage uncertainty, this BIA did not include potential cost offset from HbA1c improvements. However, several real-world studies showed an association between CGM scan rate and HbA1c control [30-32]. Moreover, a previous BIA has included cost offsets due to improved HbA1c [33].

The model omits treatment of hypoglycaemia and DKA outside of the modelled framework. It is entirely within reason that some patients experiencing hypoglycaemia or DKA may contact a healthcare professional for advice or treatment. HES data for hypoglycaemia and DKA include a column labelled “Emergency”, in which, some admissions were previously reported [22]. Suggesting that some admissions were emergencies while others were elective. This model only considers emergency admissions, elective admissions pathways were not accounted for in this BIA. Also, mild DKA and ambulance care of DKA episodes are not costed at all in this model, due to lack of data. This omission is not expected to cause a large impact on the final outcome.

Therefore, it is likely that this BIA underestimates the cost savings associated with CGM on healthcare budgets and service delivery, as well as the wider community.

## Model inputs were not validated by relevant decision-makers

As recommended by the ISPOR 2012 BIA Task Force, the “validation of the model should include at least face validity with decision-makers and verification of the calculations” [34]. Only the latter was performed. Validation of the model structure and inputs by suitable external decision-makers would have mitigated the uncertainty and limitations discussed herein. The knowledge and experience of the authors was thought to adequately satisfy this criterion.

## Rates used for hypoglycaemia and DKA

The rates of hypoglycaemia and DKA used in the SMBG group are likely to be underestimates for the NICE defined subset, i.e. those with impaired awareness, recurrent hypoglycaemia, SH or those who test ≥8 times/day [5]. The rates used represent general insulin users, not those with complications.

For CGM, the rate of reduction used in the basecase (-48.8%) is also likely to be an underestimate. The rate of reduction used in the basecase was based on the American Diabetes Association (ADA) blood glucose level to define SH in the pivotal study (<2.5 mmol/L), in line with the previous BIA in T2DM patients [10]. However, CGM patients experienced no SH in the 6-month pivotal study [29] or the 12-month extension [35], as defined in this analysis (patient requires 3^rd^ party assistance). The pivotal study may have been underpowered to discover SH events, therefore, the approach adopted was thought to limit modelling uncertainty.

Therefore, scenario 1 (CGM stops all hypoglycaemia and DKA) may be a better reflection of reality. In scenario 1, CGM was cost-saving, compared with SMBG (Figure 4 in the main paper).

## The use of prescribing data to infer proportionality

On several occasions, prescribing data were used to calculate inputs for the model. It is assumed that total prescribing volumes of the products reviewed was representative of prescribing in the NICE-eligible cohort. However, it may be that these products are disproportionately used in some patients (e.g., products to manage hypoglycaemia disproportionately prescribed in patients on basal-bolus or bolus only insulin). Due to the lack of data, the pragmatic approach adopted was thought to manage uncertainty.

Please note that raw prescribing data were used; prescribing errors, medication waste and off-label prescribing were not accounted for.

For insulin prescribing extracts, vial preparations were not included in the analysis. However, their contribution would have been minimal as they are not thought to be relevant for the NICE-eligible cohort.

## Lack of consideration for sulfonylureas

Oral anti-diabetics are recommended before the initiation of insulin in T2DM [3]. Therefore, it is likely that most T2DM patients are treated with at least one oral anti-diabetic. The risk of hypoglycaemia is generally considered to be negligible with non-insulin treatment [9], except for sulfonylureas, where hypoglycaemia is reported as a common or very common adverse effect [2]. In fact, it is reported as 0.045 PPPY, 95% CI 0.023 to 0.115 [9], which is less frequent than insulin.

The model did not consider the cumulative added risk of hypoglycaemia in patients who use both insulin and sulfonylureas. Sulfonylurea inclusion may increase the rate of hypoglycaemia episodes and improve the cost efficiency of CGM. The impact of this is expected to be limited as “the greatest [economic] burden of hypoglycaemia is associated with exogenous insulin therapy” [19]. Data to inform the number of patients using both insulin and sulfonylureas in England was not found.

## Ambulance callout rate

In the second step of the SH treatment pathway, ambulance treatment is included in 29.3% of SH cases [25]. This figure was derived from a study, which aggregated healthcare utilisation data from insulin degludec studies in T1DM and T2DM patients. In total, the study considered 536 SH episodes, of which 116 were in T2DM patients and 420 were in T1DM patients. Ambulance treatment was required in 130 T1DM and 27 T2DM SH episodes [25]. These data are detailed in Table s16.

**Table s16: Proportion of SH events that required ambulance attendance** [25]

| **Group** | **SH events** | **Events involving ambulance** |
| --- | --- | --- |
| T1DM basal-bolus | 420 | 130 (31.0%) |
| T2DM multiple daily injections | 95 | 24 (25.3) |
| T2DM basal–oral therapy | 21 | 3 (14.3%) |
| **Total** | **536** | **157 (29.3%)** |

SH: Severe hypoglycaemia, T1DM: Type 1 diabetes mellitus, T2DM: Type 2 diabetes mellitus

The primary limitation is that the figure used in the basecase better represents T1DM SH, rather than T2DM SH. Rates for T2DM were not used as they were based on a very small number of patients.

Therefore, the basecase used 29.3%; the average of all episodes, as SH treatment protocols are similar, irrespective of the type of diabetes [2]. Other insulins may require different rates of healthcare utilisation, although this is thought to be unlikely.

Furthermore, the data were derived from 15 multi-national randomised controlled trials on insulin degludec [25], which may not be representative of England’s healthcare system. The uncertainty generated by these factors is thought to be limited.

## The rate of mild hypoglycaemia incidence rate

The previously reported incidence rates were based on how many years patients had been using insulin [8]. In this study, the groups considered were T2DM insulin users <2 years and T2DM insulin users >5 years [8]. This was an observational study, which did not include T2DM patients who had been using insulin between 2 and 5 years. Therefore, calculations that utilise these figures as inputs are subject to limited uncertainty.

## The NICE resource impact tool [5] was a fundamental driver of the model

The NICE Resource Impact Template was specifically built to estimate the impact on the NICE-eligible population. As this model takes the NICE criteria for inclusion, it may not provide accurate estimates for decision-makers who wish to reimburse CGM for patients outside of the NICE guideline.

Furthermore, the NICE patient funnel [5] utilises the general population and diabetes prevalence rates to estimate the NICE-eligible population. However, data from the England and Wales National Diabetes Audit (NDA) [36] may provide diabetes patient numbers with greater accuracy as 99.3% of primary care providers provided registered patient numbers in the 2020-21 NDA [37]. As can be seen from Table s17, the NICE approach estimates the T1DM to be larger by 14% than the number of T1DM patients registered with a GP in England. In comparison, the NICE estimate for T2DM and other diabetes patients is smaller than the NDA figure by 6% (T2DM and other shown as NDA data do not show T2DM as a discrete subset).

**Table s17: Diabetes population comparison**

|  | **NICE approach** | | **National Diabetes Audit** | |
| --- | --- | --- | --- | --- |
|  | Patients | Source | Patients | Reference |
| **T1DM** | 291,542 | See Table s4 | 256,395 | [38] |
| **T2DM and other** | 3,034,802 | Used data from the patient funnel (Results, main paper) and a proportion of 92% to exclude T1DM [5] | 3,218,895 | [38] |

Please note the table shows T2DM and other as the National Diabetes Audit data do not show T2DM as a discrete subset.

T1DM: Type 1 diabetes mellitus, T2DM: Type 2 diabetes mellitus

This variance may impact the cost of using either CGM or SMBG but is unlikely to fundamentally change the CGM budget impact.

Furthermore, the reference for some lines in the NICE patient funnel was “NHSE clinical opinion”. This includes the proportion of T2DM patients on multiple daily insulin injections [5]. Clinical opinion is ranked lowest in the pyramid of evidence [39].

## SH and DKA triggered attendances in primary care

This modelling approach may over-simplify local arrangements. It could be that these medication reviews and structured education sessions are performed in hospital, before discharge, without active GP Practice involvement. As such the activity and cost would be allocated to secondary care, rather than primary care. This may change where cost savings are derived but is not expected to fundamentally change the outcomes of the model.

# REFERENCES

1. Lee W, Smith E, Chubb B, Wolden M. Frequency of blood glucose testing among insulin-treated diabetes mellitus patients in the United Kingdom. Journal of Medical Economics. 2014;17(3):167-75.

2. BNF. British National Formulary (BNF). <https://bnf.nice.org.uk> (2023). Accessed September 2023.

3. NICE. Type 2 diabetes in adults: management. NICE guideline [NG28]. <https://www.nice.org.uk/guidance/ng28/> (2022). Accessed May 2022.

4. OpenPrescribing.net. Bennett Institute for Applied Data Science, University of Oxford. <https://openprescribing.net/> (2022). Accessed September 2023.

5. NICE. Resource impact template: Type 1 & 2 diabetes in adults, children and young people - glucose monitoring. <https://www.nice.org.uk/guidance/ng28/resources> (2022). Accessed May 2022.

6. Office for National Statistics. Mid-Year Population Estimates, UK, June 2020. <https://www.ons.gov.uk/peoplepopulationandcommunity/populationandmigration/populationestimates/datasets/populationestimatesforukenglandandwalesscotlandandnorthernireland> (2021). Accessed May 2022.

7. NHS Business Services Authority. Drug Tariff for England and Wales September 2023. <https://www.nhsbsa.nhs.uk/pharmacies-gp-practices-and-appliance-contractors/drug-tariff> (2023). Accessed September 2023.

8. UK Hypoglycaemia Study Group. Risk of hypoglycaemia in types 1 and 2 diabetes: effects of treatment modalities and their duration. Diabetologia. 2007;50(6):1140-7.

9. Czech M, Rdzanek E, Pawęska J, Adamowicz-Sidor O, Niewada M, Jakubczyk M. Drug-related risk of severe hypoglycaemia in observational studies: a systematic review and meta-analysis. BMC Endocrine Disorders. 2015;15(1):57.

10. Oyagüez I, Gómez-Peralta F, Artola S, Carrasco FJ, Carretero-Gómez J, García-Soidan J, et al. Cost Analysis of FreeStyle Libre® 2 System in Type 2 Diabetes Mellitus Population. Diabetes Ther. 2021;12(9):2329-42.

11. Khunti K, Alsifri S, Aronson R, Cigrovski Berković M, Enters-Weijnen C, Forsén T, et al. Rates and predictors of hypoglycaemia in 27 585 people from 24 countries with insulin-treated type 1 and type 2 diabetes: the global HAT study. Diabetes, Obesity and Metabolism. 2016;18(9):907-15.

12. Hellmund R, Weitgasser R, Blissett D. Cost calculation for a flash glucose monitoring system for UK adults with type 1 diabetes mellitus receiving intensive insulin treatment. Diabetes Research and Clinical Practice. 2018;138:193-200.

13. NHS. Low blood sugar (hypoglycaemia). <https://www.nhs.uk/conditions/low-blood-sugar-hypoglycaemia/> (2023). Accessed December 2023.

14. Khunti K, Fisher H, Paul S, Iqbal M, Davies MJ, Siriwardena AN. Severe hypoglycaemia requiring emergency medical assistance by ambulance services in the East Midlands: a retrospective study. Prim Care Diabetes. 2013;7(2):159-65.

15. Khunti K, Fisher H, Paul S, Iqbal M, Davies MJ, Siriwardena AN. Severe hypoglycaemia requiring emergency medical assistance by ambulance services in the East Midlands, UK (poster). <https://eprints.lincoln.ac.uk/id/eprint/6252/1/EASDhypoglyaemiaposter.pdf> (2012). Accessed May 2022.

16. NHS England. NHS England. 2023-25 NHS Payment Scheme. 2023/24 prices workbook. 2023. <https://www.england.nhs.uk/publication/2023-25-nhs-payment-scheme/> (2023). Accessed September 2023.

17. NHS England. National Cost Collection data. National schedule of NHS costs. 2023. <https://www.england.nhs.uk/costing-in-the-nhs/national-cost-collection/#ncc1819> (2021/2022). Accessed September 2023.

18. JBDS-IP, Walden E, Stanisstreet D, Graveling A, Amiel S, Crowley C, et al. Joint British Diabetes Societies for Inpatient Care. The Hospital Management of Hypoglycaemia in Adults with Diabetes Mellitus. 3rd edition. <https://diabetes-resources-production.s3.eu-west-1.amazonaws.com/resources-s3/2018-05/JBDS_HypoGuidelineRevised2.pdf%2008.05.18.pdf> (2018). Accessed June 2022.

19. Parekh WA, Ashley D, Chubb B, Gillies H, Evans M. Approach to assessing the economic impact of insulin-related hypoglycaemia using the novel Local Impact of Hypoglycaemia Tool. Diabetic Medicine. 2015;32(9):1156-66.

20. Zhong VW, Juhaeri J, Mayer-Davis EJ. Trends in Hospital Admission for Diabetic Ketoacidosis in Adults With Type 1 and Type 2 Diabetes in England, 1998-2013: A Retrospective Cohort Study. Diabetes Care. 2018;41(9):1870-7.

21. Zhong VW, Juhaeri J, Mayer-Davis EJ. Supplementary Data. Trends in Hospital Admission for Diabetic Ketoacidosis in Adults With Type 1 and Type 2 Diabetes in England, 1998-2013: A Retrospective Cohort Study. Diabetes Care. 2018;41(9):1870-7.

22. NHS Digital. Hospital Admitted Patient Care Activity, 2019-20: Diagnosis. <https://digital.nhs.uk/data-and-information/publications/statistical/hospital-admitted-patient-care-activity/2019-20> (2020). Accessed May 2022.

23. JBDS-IP, Claydon A, Dyer P, Evans P, Khan A, Kilvert A, et al. Joint British Diabetes Societies for Inpatient Care. The Management of Diabetic Ketoacidosis in Adults. <https://www.diabetes.org.uk/professionals/position-statements-reports/specialist-care-for-children-and-adults-and-complications/the-management-of-diabetic-ketoacidosis-in-adults> (2021). Accessed June 2022.

24. Foos V, Varol N, Curtis BH, Boye KS, Grant D, Palmer JL, et al. Economic impact of severe and non-severe hypoglycemia in patients with Type 1 and Type 2 diabetes in the United States. Journal of Medical Economics. 2015;18(6):420-32.

25. Heller SR, Frier BM, Hersløv ML, Gundgaard J, Gough SCL. Severe hypoglycaemia in adults with insulin‐treated diabetes: impact on healthcare resources. Diabetic Medicine. 2016;33(4):471-7.

26. Farmer AJ, Brockbank KJ, Keech ML, England EJ, Deakin CD. Incidence and costs of severe hypoglycaemia requiring attendance by the emergency medical services in South Central England. Diabetic Medicine. 2012;29(11):1447-50.

27. Brod M, Christensen T, Thomsen TL, Bushnell DM. The impact of non-severe hypoglycemic events on work productivity and diabetes management. Value Health. 2011;14(5):665-71.

28. Pawaskar M, Witt EA, Engel SS, Rajpathak SN, Iglay K. Severity of hypoglycaemia and health‐related quality of life, work productivity and healthcare costs in patients with type 2 diabetes in Europe. Endocrinol Diabetes Metab. 2018;1(2):e00011.

29. Haak T, Hanaire H, Ajjan R, Hermanns N, Riveline J-P, Rayman G. Flash Glucose-Sensing Technology as a Replacement for Blood Glucose Monitoring for the Management of Insulin-Treated Type 2 Diabetes: a Multicenter, Open-Label Randomized Controlled Trial. Diabetes Ther. 2017;8(1):55-73.

30. Dunn TC, Xu Y, Hayter G, Ajjan RA. Real-world flash glucose monitoring patterns and associations between self-monitoring frequency and glycaemic measures: A European analysis of over 60 million glucose tests. Diabetes Research and Clinical Practice. 2018;137:37-46.

31. Calliari LEP, Krakauer M, Vianna AGD, Ram Y, Barbieri DE, Xu Y, et al. Real-world flash glucose monitoring in Brazil: can sensors make a difference in diabetes management in developing countries? Diabetol Metab Syndr. 2020;12:3.

32. Gomez-Peralta F, Dunn T, Landuyt K, Xu Y, Merino-Torres JF. Flash glucose monitoring reduces glycemic variability and hypoglycemia: real-world data from Spain. BMJ Open Diabetes Res Care. 2020;8(1):e001052.

33. Frank JR, Blissett D, Hellmund R, Virdi N. Budget Impact of the Flash Continuous Glucose Monitoring System in Medicaid Diabetes Beneficiaries Treated with Intensive Insulin Therapy. Diabetes Technology & Therapeutics. 2021;23(S3):S36-S44.

34. Sullivan SD, Mauskopf JA, Augustovski F, Jaime Caro J, Lee KM, Minchin M, et al. Budget Impact Analysis—Principles of Good Practice: Report of the ISPOR 2012 Budget Impact Analysis Good Practice II Task Force. Value in Health. 2014;17(1):5-14.

35. Haak T, Hanaire H, Ajjan R, Hermanns N, Riveline J-P, Rayman G. Use of Flash Glucose-Sensing Technology for 12 months as a Replacement for Blood Glucose Monitoring in Insulin-treated Type 2 Diabetes. Diabetes Ther. 2017;8(3):573-86.

36. NHS Digital. Report 1: Care Processes and Treatment Targets 2019 - 20, Full Report. National Diabetes Audit 2019-20 Full Report 1, Open Data - Registrations. <https://digital.nhs.uk/data-and-information/publications/statistical/national-diabetes-audit/report-1-care-processes-and-treatment-targets-2019---20> (2021). Accessed April, 2022.

37. NHS Digital. Report 1: Care Processes and Treatment Targets 2020-21, Full Report. Executive Summary: Audit Participation. <https://digital.nhs.uk/data-and-information/publications/statistical/national-diabetes-audit/core-report-1-2020-21/audit-participation> (2022). Accessed July 2022.

38. NHS Digital. National Diabetes Audit (NDA) 2020-21 Data Release for England, Clinical Commissioning Groups and GP practices. <https://digital.nhs.uk/data-and-information/publications/statistical/national-diabetes-audit/report-1--care-processes-and-treatment-targets-2020-21-underlying-data> (2021). Accessed Febuary 2022.

39. Burns PB, Rohrich RJ, Chung KC. The Levels of Evidence and their role in Evidence-Based Medicine. Plast Reconstr Surg. 2011;128(1):305-10.
